# Tyrosinase Cross-Linked PEG Hydrogels with DAT and DATT as Artificial Substrates: Design, Structure, and Functions

**DOI:** 10.1021/acs.biomac.5c01929

**Published:** 2026-01-26

**Authors:** Miroslava Racheva, Javier Basalo Lourido, Enise Ece Gurdal, Martin Herbst, Seyhmus Bayar, Daniela Radzik, Elen Bähr, Constanze Zwies, Axel T. Neffe, Markus Pietzsch, Andreas Lendlein, Christian Wischke

**Affiliations:** † Institute of Active Polymers, Helmholtz-Zentrum Hereon, Kantstrasse 55, 14513 Teltow, Germany; ‡ Institute of Pharmacy, Martin-Luther-University Halle-Wittenberg, Kurt-Mothes-Strasse 3, 06120 Halle, Germany; § European Center of Just Transition Research and Impact-Driven Transfer (JTC), Martin-Luther-University Halle-Wittenberg, 06099 Halle, Germany

## Abstract

Enzymes such as oxidases are sustainable tools for hydrogel synthesis, but complex competing reactions have limited the mechanistic understanding and biomedical applications of these materials. Guided by molecular docking and MM-GBSA calculations, we identified two artificial substrates, desaminotyrosine (DAT) and desaminotyrosyltyrosine (DATT), that were experimentally more efficiently converted by mushroom tyrosinase (mTyr) than the natural substrate tyrosine. These substrates were used to synthesize hydrogels from DAT/DATT-functionalized star-shaped oligoethylene glycol (sOEG). Model reactions elucidated the chemical nature and functionality of the hydrogel netpoints. Material properties were systematically investigated depending on sOEG molecular weight (5, 10, 20 kDa), substrate type, and mTyr concentration. Functional mesh sizes and controlled release functions were investigated with fluorescent dextrans (4–500 kDa) and heparin. Cell culture studies with L929 fibroblasts and THP-1 monocytes suggested inertness of the material. These findings provide fundamental insight into mTyr-catalyzed hydrogel formation and support further exploration for in situ hydrogel synthesis.

## Graphical abstract

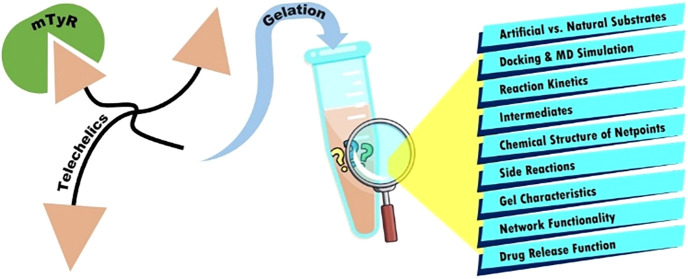

## 1 Introduction

Enzymes deserve attention not only as a renewable source of catalytic agents for synthetic biology and the stereoselective synthesis of fine chemicals and pharmaceutical drugs,^1–4^ but also in material science. The spectrum of enzymatic reactions to create functional materials spans from surface chemistry, e.g., for coating purposes^5,6^ to the in situ formation of bulk material.^7–9^

In order to synthesize materials—here especially hydrogels—via biocatalysis, precursor materials (oligomers or polymers) are needed that later form the network structure. These telechelic precursors must contain moieties that will be subject to enzymatic conversion. In principle, various biopolymers such as alginate or gelatin^10,11^ as well as synthetic polymers based on, e.g., 2-hydroxyethyl methacrylate,^12^ *N*-vinyl caprolactam,^13^ or poly/oligoethylene glycol^14^ have been considered as backbone material for enzymatic cross-linking. Out of these, oligoethylene glycol (OEG) is one of the most common materials for hydrogel synthesis due to its well-defined structure and excellent solubility.^15–19^ Using OEG (low molecular weight variant of poly­(ethylene glycol)) is advantageous, as short-chain oligomers allow the formation of hydrogel networks with low segment length between the netpoints, which is necessary to control the hydrogel swelling and the diffusion of embedded payloads. To enable the enzymatic cross-linking of OEG to a hydrogel network, suitable chemical moieties (natural or artificial enzyme substrates) have to be coupled to OEG. For this coupling, the end groups of the OEG chains can be used.

During the enzymatic cross-linking reaction, the network telechelics will be in an aqueous environment, which can be considered to be a semidiluted state with some entanglements of individual chains. Under these conditions, the substrates (end groups of polymers) will be restricted in their diffusion radii. The probability of substrate access to the enzyme and product–product interaction can be increased when a higher number of chain ends (substrates) are present in the precursors. This can be achieved by using branched (e.g., star-shaped) rather than linear oligomers. Additionally, the atoms at the branching points of the telechelics will later serve as additional netpoints in the hydrogel networks.

A number of oxidoreductase enzymes, namely horseradish peroxidase (HRP), glucose oxidase, and laccase, have been explored for the enzymatic material synthesis.^20^ For instance, HRP allows a fast coupling of various aromatic moieties, including phenols, phenylamines, indoles as well as sulfonates and similar structures through substrate conversion to radicals using H_2_O_2_ as a (potentially toxic) oxidation cofactor.^21^ However, the low substrate specificity of HRP may also result in the conversion of aromatic moieties of potential hydrogel payloads or medium components, thus limiting the predictability of network structures.^22^ A higher substrate specificity can be found in tyrosinase, which catalyzes the oxidation of the natural substrates l-tyrosine and l-DOPA (l-3,4-dihydroxyphenylalanine), which is the rate-determining step in the physiological formation of melanin pigments. Tyrosinases are ubiquitously distributed, with mushroom tyrosinase (mTyr) being a well accessible, sustainable catalyst that has a track record in biomedical material syntheses.^7,23–27^ mTyr operates through four different oxidation stages involving the binding and consumption of molecular oxygen as cosubstrate,^28^ which is soluble in biological media and should not raise toxicity concerns.^9^ Thus, mTyr was selected in this study to design functional hydrogel materials.

However, multiple challenges are associated with a biocatalytic hydrogel synthesis. Besides the structural requirement that the precursor’s end group (substrate) needs to access the active site of the enzyme and can be converted,^29^ it is important to experimentally prove that this reaction eventually leads to cross-linking to hydrogel networks. Another important aspect is to understand the structures that are eventually being formed in the material as a consequence of the enzymatic conversion of certain moieties. The importance of this task can be seen in bulk hydrogels promoted for medical applications like tissue engineering.^30^ Here, regulatory requirements to assess, e.g., the necessary safety of the involved chemical structures can only be fulfilled if the formed structures can be elucidated in detail. The oxidoreductases, which are typically selected for biomaterial synthesis,^1^ typically create free radical species.^20,22^ In consequence, different types of intermediates can be formed that can enter into competing (spontaneous) cross-linking pathways, e.g., in the case of catechols.^31,32^ The products of such material syntheses cannot be directly analyzed by solution-based analytical techniques as they are cross-linked networks, i.e., not freely soluble.^33,34^ Furthermore, literature suggests that tyrosinase accepts a number of hydroxycinnamic acid derivatives^35,36^ as well as hydroxybenzoic acid derivatives and further phenolic compounds^37–40^ as substrates besides tyrosine, while it is also known that some minor structural variations of such molecules can produce inhibitors rather than substrates of tyrosinase.^36,41^ Accordingly, it remains a challenge to select suitable substrates and provide a structural analysis of enzymatically cross-linked hydrogels.^42,43^

Key steps in this work (Figure 1) will be (i) the identification of suitable substrate molecules based on conversion kinetics, which subsequently should be coupled to four-arm star-shaped OEG-amine (sOEG-NH_2_) precursors as network telechelics. To elucidate cross-linking mechanisms and characteristics, (ii) model reactions will be performed to investigate the substrate conversion and formed intermediates, the number of substrates coupled in formed adducts, the structure of formed multimers, and the contribution of different competitive cross-linking pathways toward product formation. Finally, (iii) structure–function relationships in synthesized bulk hydrogel networks will be evaluated, including the capability to rate the diffusion of incorporated molecules.

**Figure 1:**
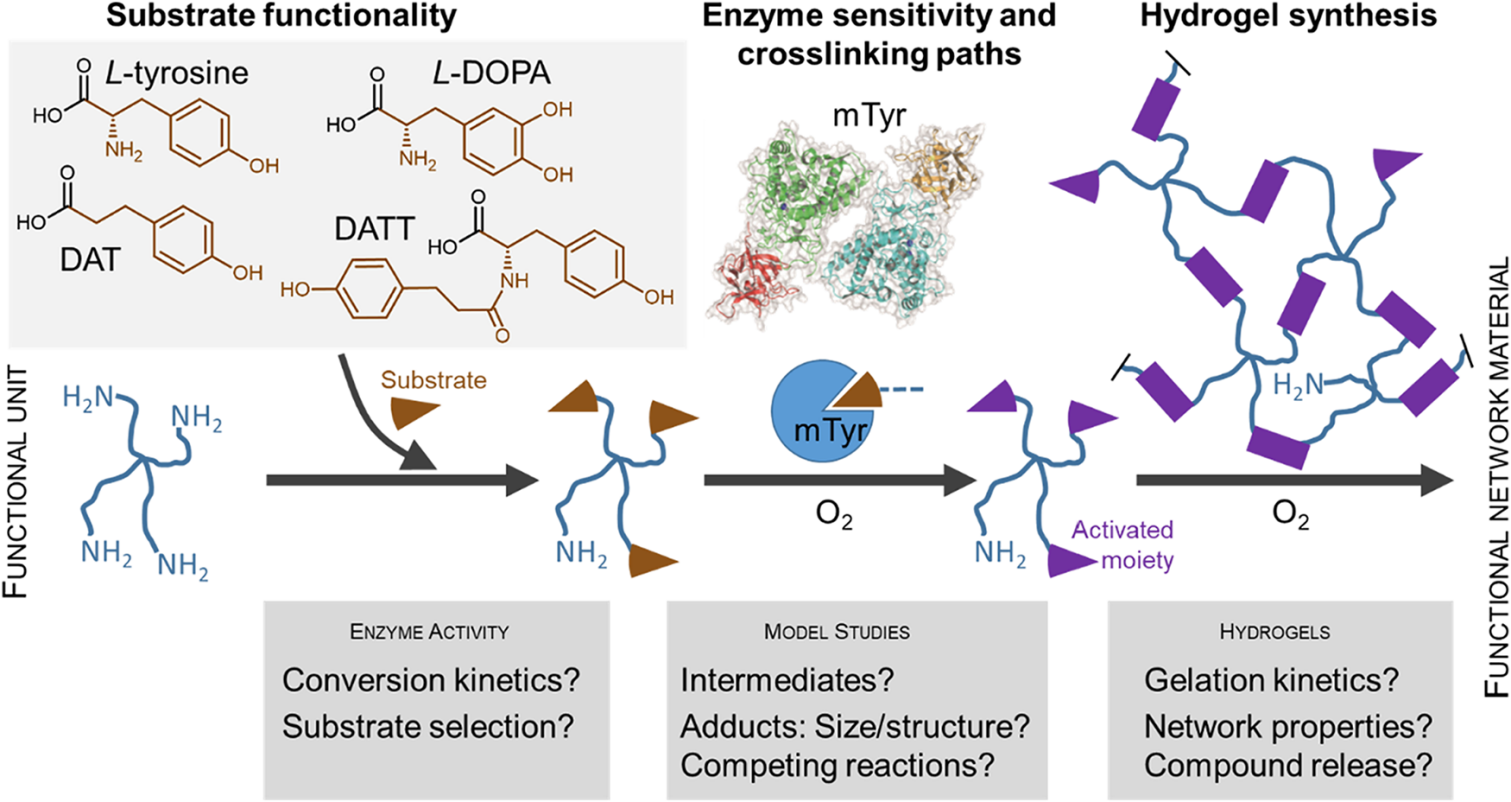
Schematic representation of the study concept. The evaluation of different chemical moieties as enzymatic substrates provides selection criteria for coupling such molecules to hydrophilic functional units (telechelics; sOEG-NH_2_) as required to build network materials. Model studies of mTyr reaction with substrates explore the cross-linking paths after enzymatic conversion of substrates to activated moieties. Hydrogels obtained through enzymatic cross-linking are characterized regarding structure–function relationships.

## 2 Experimental Section

### 2.1 Materials

Tyrosinase from mushroom (mTyr) was purchased from Sigma-Aldrich Chemie GmbH (Steinheim, Germany) and used without further purification. Stock solutions of mTyr were prepared in PBS buffer, aliquoted, frozen at −26 °C, and the respective number of aliquots were thawed and further diluted with oxygen-saturated PBS as needed for the experiments. DAT (desaminotyrosine; 3-(4-hydroxyphenyl)­propionic acid), DAT methylester, l-tyrosine, l-DOPA, MBTH (3-Methyl-2-benzothiazolinone hydrazone hydrochloride; Fluka analytical grade), and heparin (heparin sodium salt from porcine intestinal mucosa) were purchased from Sigma-Aldrich Chemie GmbH (Steinheim, Germany). Oligomer precursors for functionalization, i.e., sOEG-NH_2_·HCl (*M*_n_ = 5, 10, or 20 kDa) and NH_2_-lOEG_OMe_ (Methoxy PEG Amine, HCl Salt, *M*_n_ = 2 and 10 kDa) were obtained from Jen Kem Technology (Beijing, China) and the monodisperse oligomer NH_2_-lmOEG_OMe_ (MeO-dPEG(48)-NH_2_) from Iris Biotech GmbH (Marktredwitz, Germany). FITC-labeled dextrans (FITC-Dex) with *M*_n_ = 4, 20, 40, 70, 150, and 500 kDa were purchased from TdB Consultancy AB (Uppsala, Sweden). The solvents were of ACS grade when used for synthesis and of HPLC grade for chromatographic purposes.

For cell culture, resazurin and Triton-X-100 from Sigma-Aldrich/Merck KGaA (Darmstadt, Germany) were used. 2-p-Iodophenyl-3-p-nitrophenyl tetrazolium chloride (INT), β-nicotinamide adenine dinucleotide sodium (β-NAD), and 1-methoxyphenazine methosulfate (MPMS) were from MedChemExpress LLC (Monmouth Junction, NJ, USA). Dimethyl sulfoxide (DMSO) was purchased from CHEMSOLUTE/Th. Geyer GmbH & Co.KG (Renningen, Germany). l-(+)-lactic acid and Tris–HCl were bought from Carl Roth GmbH + Co. KG (Karlsruhe, Germany). LPS, QUANTI-Blue reagent and buffer were purchased from Invivogen (Toulouse, France). PBS, DMEM and RPMI medium, blocking reagents, Alexa Fluor labeled secondary antibodies and the CD86 antibody were from Gibco/Thermo Fisher/Life Technologies Ltd. (Paisley, U.K.). CD11c, CD45, and CD206 antibodies and the Live/Dead staining reagent for flow cytometry were from Miltenyi Biotec, while NF-κB and NLRP3 antibodies were from Cell Signaling and Proteintech, respectively. CellTiter 96 Aqueous Non-Radioactive Cell Proliferation Assay (MTS assay) was from Promega Corporation (Madison, WI, USA).

### 2.2 Docking Studies

Docking studies were carried out using Schrödinger’s software (version 2021.3) with the docking program Glide, using the X-ray crystal structure of mushroom tyrosinase (PDB ID: 2y9w)^44^ from the Protein Data Bank (PDB).^45^ Docking studies were performed for both the free ligands (tyrosine, DAT, and DATT) and their OEG_6_-amide conjugates. In Glide, the data were prepared using Schrödinger’s Protein Preparation Wizard with default settings.^46^ Protonation states were assigned at pH 7.0 using the PROPKA tool in Schrödinger. Crystallographic water molecules were removed, and the protonation state of the hydroxide ion was manually corrected to OH^–^, as the automated procedure had protonated it to water. Hydrogen-bond geometries were optimized, and the structure was energy-minimized using the OPLS_2005 force field under Schrödinger’s default settings.

For receptor grid generation, the binding site was defined to include two copper ions and one hydroxide ion, along with key residues H85, H259, N260, F264, and V283. Ligand conformers (*n* = 64 per compound) were generated using ConfGen (Schrödinger Release 2021–3). Docking was carried out using Glide in standard precision (SP) mode,^47^ with metal constraints applied to account for coordination interactions.

### 2.3 MM-GBSA Calculations

MM-GBSA (Molecular Mechanics with Generalized Born and Surface Area Solvation) calculations were carried out using Schrödinger’s Prime MM-GBSA module^48^ to estimate the binding free energies of the protein–ligand complexes. The VSGB solvation model and OPLS_2005 force field were used for all calculations. Local flexibility was allowed within 3 Å of the ligand to account for side-chain adjustments. All other parameters were set to default values.

### 2.4 Molecular Dynamics Simulations

Molecular dynamics (MD) simulations of the mTyr-DATT, -DAT, and -Tyr complexes were performed using the Desmond simulation package. System preparation employed the SPC (simple point-charge) water model and the OPLS_2005 force field. To neutralize the mTyr structure, 15 Na^+^ ions were added, along with a 0.15 M NaCl buffer solution. The system was enclosed in an orthorhombic box with a volume of 433,598 Å^3^. Simulations were conducted at 300 K and 1.013 bar for a total duration of 20 ns. Trajectories were recorded every 20 ps, yielding approximately 1000 frames for subsequent analysis. MD simulations of mTyr complexes with OEG_6_-DAT, OEG_6_-DATT, and OEG_6_-Tyr (all modeled as conjugates of amine-OEG_6_) were performed analogously to the free substrates.

For the analysis of phenol clustering, star-shaped sOEG_6_ precursors (each arm with six repetitive units) functionalized with DAT, DATT, and tyrosine were constructed in MS Maestro (Materials Science Suite, Schrödinger, LLC, New York, NY, 2025). The polymers were simulated in explicit water under the same thermodynamic conditions as the enzyme-bound systems for 20 ns. Solvent-accessible surface areas (SASA) were calculated for all phenolic heavy atoms and their hydroxyl hydrogens using a 1.4 Å probe. SASA values were extracted every 20 ps across the full trajectory. For each system, mean ± standard deviation was determined for individual phenolic groups to estimate solvent exposure and the extent of PEG-induced shielding.

### 2.5 Substrate Conversion Kinetics

The suitability of substrates for conversion by mTyr was determined by the MBTH assay at 25 °C.^49^ 20 μL of mTyr solution in PBS (55 nM), 200 μL of substrate solution in water (l-tyrosine, l-Dopa, DAT, DATT; clear 5 mM solutions), and 780 μL of MBTH (Besthorn’s hydrazine; 5.12 mM) in PBS/O_2_ supplemented with 2.74% (w/w) dimethylformamide (DMF) were mixed in PMMA cuvettes, leading to a 4-fold molar excess of MBTH. The enzyme activity was calculated from the maximum slope of the linear part of the absorption curve at 505 nm after a typical initial lag phase, using the extinction coefficient of 38,000 M^–1^·cm^–1^ as reported for MBTH-quinones.^50^

### 2.6 Synthesis of Model Compounds and Network Precursors

DAT was used as received and DATT was synthesized by a previously established procedure.^51^

The synthesis of DAT­(T)-sOEG from sOEG-NH_2_ (0.5 mmol) was performed in 80 mL *N*-methylpyrrolidone (NMP) at r.t. for 17 h via EDC/NHS chemistry (DAT­(T) 3.5 mmol, EDC·HCl 4 mmol, NHS 4.5 mmol, DIPEA 12 mmol) with subsequent purification by precipitation in cold hexane:ethyl acetate (1:1), filtration, washing with ethyl acetate and freeze-drying.^52^

The DAT functionalization of linear OEG was performed (i) for 10 kDa NH_2_-lOEG_OMe_ (0.2 mmol) by EDC/NHS coupling (DAT 0.4 mmol, EDC·HCl 0.5 mmol, NHS 0.6 mmol, DIPEA 0.8 mmol) in 40 mL NMP in an ice bath followed by precipitation and dialysis (3.5 kDa cutoff), (ii) for 2 kDa NH_2_-lOEG_OMe_ by EDC/NHS coupling in dichlormethane (17 h, r.t.) followed by extraction, filtration through basic Al_2_O_3_, drying, and further purification by preparative HPLC on a PS/DVB column, and (iii) for NH_2_-lmOEG_OMe_ (0.23 mmol) with a TBTU-based coupling (DAT 0.54 mmol, TBTU 0.58 mmol, DIPEA 0.82 mmol) in 4 mL DMF at r.t. for 3 d, followed by filtration through basic Al_2_O_3_, drying, and further purification by preparative HPLC on a PS/DVB column. All reactions were performed under a nitrogen atmosphere with stirring.

Samples characterization included ^1^H NMR spectrometry (Advance DRX 500 mHz, Bruker), ATR-FTIR spectrometry (Nicolet 6700, ThermoScientific), and MALDI-ToF mass spectrometry (ultrafleXtreme, Bruker) with 2,5-dihydroxybenzoic acid (DHB) as a matrix.

### 2.7 Model Reactions and Analyses

Information on the respective concentrations of mTyr and other reactants as well as the number of independent experiments are reported in the display items and legends.

Linear DAT monofunctionalized OEG’s were dissolved in PBS (saturated with O_2_) at pH 7.4 and mixed with a mTyr stock solution in PBS/O_2_, typically at a volume ratio of 1:1. For the different experiments, different oligomer and mTyr concentrations were used as indicated with the data. For UV/vis spectrometry (Cary 50, Varian; equipped with thermostate at 25 ± 0.5 °C), the reaction mixtures were monitored online by wavelength scans after addition of mTyr in case of low DAT-lOEG 10 kDa concentration (6.25 mg·mL^–1^). For experiments with higher reactant concentration (50 mg·mL^–1^), aliquots were diluted 50-fold before immediate measurements. ATR-FTIR analyses (Nicolet 6700, ThermoScientific) of substrate conversion of DAT-lOEG 2 kDa were performed after incubation with mTyr in PBS/O_2_ for 7 d at r.t., followed by drying to eliminate dominating signals of water.

For UHPLC-ESI-MS analyses (UPLC-Synapt G2-S HDMS, Waters) of substrate conversion, DAT-lmOEGoMe 2 kDa were incubated with mTyr for 24 h and the reaction mixture was separated on a C18 RP column with a water/acetonitrile gradient (solvents with +0.1% formic acid) over 25 min along with ESI-MS detection.

^1^H NMR analysis of reaction products was based on signal prediction in MestReNova software. Experimental data were collected for DAT methyl ester and DAT-lOEG_OMe_ 2 kDa. For DAT-lOEG_OMe_ 2 kDa, the reaction was performed in D_2_O as solvent and the samples were analyzed without further processing. For DAT methyl ester, the reaction was performed in H_2_O, as the product precipitated and water was removed by freeze-drying. The product was partially soluble in MeOH-d4 and the soluble fraction was subjected to NMR analysis (Advance DRX 500 MHz, Bruker).

To estimate the number of chain ends coupled in netpoints, DAT-lOEG_OMe_ 10 kDa was reacted with mTyr in PBS/O_2_ and subsequently prepared directly for MALDI-ToF MS (ultrafleXtreme, Bruker). DAT methyl ester was incubated with mTyr in H_2_O/O_2_; the product precipitate was isolated, freeze-dried, and analyzed. Sample preparation for analysis included the mixing of samples at ratios of 1:3 to 1:10 with DHB solutions (ACN/H_2_O + 0.1% TFA) on the MALDI target and drying under a fume hood. Only signals with a signal-to-noise ratio >6 were considered for analysis and the occurrence of the signal is reported as the AUC of the signal relative to the strongest signal of the spectrum. To determine potential contributions of nucleophilic NH_2_ end groups of telechelics in the overall network formation, DAT-lmOEG_OMe_ and NH_2_-lmOEG_OMe_ (each 2 kDa) were mixed at a 1:1 molar ratio in PBS/O_2_ for reaction with mTyr, followed by MALDI sample preparation and analysis after 24 h. The assignment of MALDI-MS signals was performed by combinatory calculations based on monoisotopic masses of potential structural motifs.

### 2.8 Hydrogel Synthesis and Analysis

Bulk hydrogel synthesis was conducted in Petri dishes (larger film samples), 1.5 mL Eppendorf tubes (tube tilting assay), or microtiter plates (diffusion/release experiments), typically by mixing equal volumes of O_2_-saturated mTyr and DAT­(T)-sOEG solutions in PBS at r.t. overnight.

The gelation kinetics were investigated by oscillatory rheometry (Physica MCR 301, Anton Paar) at 25 °C with a cone–plate geometry (25 mm; *f* = 1 Hz, γ = 0.1%). The gelation time, *t*_gel_, was determined by the crossover of tangents adjusted to the curve profile of the η*.

For determining *G* and *Q* according to eqs 1 and 2, gels as synthesized were dried, balanced (*m*_0_), extracted for 24 h first in PBS and then in water with repeated buffer change at r.t., and then either freeze-dried and balanced (*m*_ex_) or swollen in PBS at 37 °C for 24 h, blotted with paper, and balanced (*m*_sw_). The density of PBS ρ*_p_* was 1.0052 g·cm^–3^. The densities of the dry polymer networks ρ*_p_* were individually determined with an Ultra Pycnometer (Quantachrome, Odelzhausen, Germany) and used for the calculations.

$$G\;\left(\mathrm{wt}\;\%\right)=\frac{m_{\mathrm{ex}}}{m_{0}}\cdot 100$$ 1

$$Q\;\left(\mathrm{vol}\;\%\right)=1+\rho_{p}\left(\frac{m_{\mathrm{sw}}}{m_{d}\cdot \rho_{s}}-\frac{1}{\rho_{s}}\right)\cdot 100$$ 2

Rheological characteristics of preformed, extracted gel slabs were studied with a plate–plate geometry (*d* = 25 mm; gap 1.4–1.7 mm, normal force 0.1 Pa) at 37 °C. After determining the linear viscoelastic region by frequency (*f* = 0.05–50 Hz at fixed γ = 0.05%) and amplitude (γ = 0.01–100% at fixed *f* = 1 Hz) sweep measurements, all further experiments were conducted at *f* = 1 Hz and γ = 0.1% for 30 min to calculate G*′* from >100 data points.

### 2.9 Diffusion and Release Studies

Gels of a thickness of 2.5 mm, each loaded with 50 μg of FITC-dextran of the respective *M*_n_, were synthesized overnight at r.t. at the bottom of 96-well microtiter plates with light protection. The gels were covered with 250 μL PBS, out of which 100 μL were sampled and replaced with fresh buffer at each time point (37 °C, mild agitation, light protection). The released FITC-dextran was determined fluorometrically (Ex 490 nm, Em 520 nm; Infinite 200Pro, Tecan). The cumulative release profiles were fitted in Origin 9.6 by a power function (*y* = *a*·*t* ^0.5^), where the fitting parameter *a* was used to calculate the diffusion coefficients in the gel *D_g_* according to an empirical model (eq 3).^53^

$$\frac{M_{t}}{M_{\infty }}≅2.12\cdot {\left(\frac{D_{g}}{\pi \cdot l^{2}}\right)}^{0.5}\cdot t^{0.5}$$ 3

For investigating heparin release, gels of 200 μL volume with 500 μg heparin each were synthesized at different polymer concentrations in 24-well plates (overnight, r.t.), covered with 700 μL PBS, and subjected to incubation (37 °C; mild agitation) and sampling of 200 μL medium at different time points (replaced with fresh medium). Fluorometric quantification of heparin was conducted after hydrolysis and derivatization with 3,5-diaminobenzoic acid.^54^

### 2.10 Biological Studies

For evaluation of cell responses, hydrogels were prepared by mixing equal volumes of 10 wt % DAT-sOEG 10 kDa solution in PBS (saturated with O_2_) with mTyr solution (1000 U/mL) in PBS, leading to 5 wt % gels with 500 U/mL mTyr. Depending on the size of the cell culture plates, 32 μL (96-well plates) or 350 μL (12-well plates) of each stock solution were mixed, and the plates were gently shaken for 24 h at r.t. to allow complete gelation, forming 1–2 mm thick films. The gels were subsequently equilibrated with 100 μL of culture medium for 48 h with one intermediate medium change.

L929 cells were cultivated in DMEM (Gibco/Thermo Fisher/Life Technologies Ltd.; Paisley, U.K.) with 10% fetal bovine serum and 4 mM Glutamin (Biowest SAS; Nuaillé, France). THP1 as well as THP1-Blue cells (Invivogen, Toulouse, France) were cultivated in RPMI 1640 + GlutaMAX (Gibco/Thermo Fisher/Life Technologies Ltd.; Paisley, U.K.) with 10% fetal bovine serum (Biowest SAS; Nuaillé, France). All cells were kept at 37 °C and 5% CO_2_ in a C170 incubator (Binder GmbH; Tuttlingen, Germany).

For cytotoxicity testing, L929 cells were seeded at a density of 5000 cells/well directly onto the gels or treated with the corresponding amount of soluble PEG-DAT and mTyr for 48 h. As a second cell type of investigation, 40,000 THP1-Blue cells/well were studied in 96-well plates. Untreated cells served as a negative control in both cases. A dead cell control (LC; 0% viability) was prepared by exposing untreated cells to 1.2% Triton-X-100 (LDH assay) or 1 mM copper chloride (MTS assay). 50 μL supernatant of all samples (including the lysis control) was collected from each well and transferred to another 96-well plate for LDH measurements. The remaining cells and supernatant were used for MTS assay.

For the LDH assay, 50 μL LDH-substrate solution (54 mM lactic acid, 1.3 mM β-NAD^+^, 0.66 mM INT, and 0.028 mM MPMS in 0.2 M Tris-HCl buffer pH 8.2)^55^ was added to 50 μL of the cell-free supernatant. After incubation for 15 min, the absorbance was measured with a Cytation 5 instrument (Agilent Technologies, Inc.; Santa Clara, United States). The cytotoxicity was calculated according to eq 4 using the absorbance *A* of the different samples.

4 $$\mathrm{cytotoxicity}\;\left(\%\right)=\frac{A_{\mathrm{test}}-A_{\mathrm{untreated}}}{A_{\mathrm{LC}}-A_{\mathrm{untreated}}}\cdot 100\%$$

For the MTS assay, the reagent was prepared according to the manufacturer’s procedure. 20 μL of assay reagent was added to 100 μL of cell suspensions of L929 cells and incubated for 3 h. 100 μL of supernatants were transferred to a fresh 96-well plate and the absorbance was measured with the Cytation 5. The cytotoxicity was calculated according to eq 4.

A potential activation of the NF-κB pathways of THP1-Blue cells was investigated via the QANTI-Blue assay using 10 μL of cell-free supernatant harvested along with the sampling for the LDH assay (details above). The QUANTI-Blue reagent was prepared according to the manufacturer’s procedure. 90 μL QB-solution was added to 10 μL sample and incubated for 1 h. The absorbance was measured at 640 nm using the Cytation 5. As a positive control, cells treated with 0.1 μg/mL LPS were used.

Immunofluorescence staining and flow cytometry analyses were conducted after seeding 1 × 10^6^ THP-1 cells onto hydrogels in 12-well plates and incubating them in 1 mL of RPMI 1640 medium supplemented with GlutaMAX and 10% fetal bovine serum at 37 °C with 5% CO_2_ for 1 d. Cells were collected in a 2 mL tube and spun at 5000 rpm for 5 min. The excess supernatant was discarded and the cells were transferred to a 96-well plate.

For immunofluorescence imaging, cells were washed with PBS and then fixed with 2% paraformaldehyde for 10 min at r.t. The samples were permeabilized with 0.1% Triton X-100 for 5 min at r.t., exposed to a blocking solution (2% NGS, 2% BSA, 0.1% Triton X-100) for 1 h at r.t., and incubated with NF-κB and NLRP3 antibodies overnight at 4 °C. Subsequently, the samples were washed with PBS and incubated with Alexa Fluor 488 and Alexa Fluor 647 -labeled secondary antibodies for 2 h at r.t., washed 3 times with PBS, and resuspended in 100 μL PBS. A 10 μL aliquot of each sample was transferred into a glass 96-well plate (Greiner) containing 90 μL PBS. The plate was centrifuged at 500*g* for 5 min and the supernatants were discarded. The plate was dried for 2 h at r.t. and treated with 20 μL mounting medium containing DAPI. Imaging was performed using a Leica TCS SP8 X laser scanning confocal microscope. The mean fluorescent intensity of the whole images was measured in Fiji software and normalized to the DAPI staining.

For flow cytometric detection of surface markers, cells were incubated with FcR blocking reagent (Miltenyi Biotec) at 4 °C for 10 min. Samples were centrifuged at 500*g* for 5 min at 4 °C, and the supernatant was discarded. Cells were stained with conjugated antibody mixes, including CD11c, CD45, CD86, and CD206 for 30 min at 4 °C. Cells were stained with fixable LIVE/DEAD (Miltenyi Biotec) for 15 min at 4 °C. Cells were resuspended with staining buffer (BioLegend, 420201), then measured on Fortessa (BD Biosciences), and the data were analyzed in FCSExpress.

### 2.11 Statistical Analysis and Experimental Errors

The reported values of quantitative analysis in the display items are given as mean ± SD based on independent repetitions with *n* as stated in the figures. The errors of experimental and analytical methods are estimated to be typically in the range of 1–5%.

## 3 Results and Discussion

### 3.1 Selection of Potential Substrates

Taking tyrosine as the natural substrate of mTyr as a lead motif, candidate substrates are selected in the first part of this study (Figure 1). It is obvious that the phenol, the amine group, and the carboxyl moiety are characteristic structural elements of tyrosine. Some of these groups were prerequisites in this study: The carboxyl function of tyrosine can react with amines to form amide linkages, which was used for later coupling to sOEG polymer network precursors. The phenolic group is a typical substrate of mTyr. The active site of the enzyme can adapt three different oxidation states: *met*-tyrosinase, *oxy*-tyrosinase, and *deoxy*-tyrosinase.^28,50,56^ Only *oxy*-tyrosinase contains molecular oxygen bound in its active center, which is necessary for converting tyrosine, a phenolic substrate, into dopaquinone, an *o*-quinone, via its monooxygenase activity (monophenolase activity).^28^ A fraction of the formed dopaquinone, however, will cyclize spontaneously to an indoline derivative (cyclodopa) by binding the α-amino group of dopaquinone to the 6*′* carbon of the aromatic ring,^28^ adding undesired structural complexity to the system. Furthermore, as suggested earlier,^57^ π–π stacking interaction of substrates with a histidine residue supports their reorientation in the active center, eventually allowing the desired phenol hydroxylation to an *o*-quinone structure. The α-amine group of tyrosine may not be required for this entry or orientation to the active center. Removal of the α-amine function of tyrosine results in desaminotyrosine (DAT, see Figure 1), a candidate artificial substrate that was hypothesized to preserve substrate activity while excluding cyclodopa formation. Desaminotyrosyltyrosine (DATT) was considered as a second candidate substrate. As DATT contains two phenol residues (see Figure 1), the chances of conversion of at least one of the phenolic groups by the monooxygenase activity of mTyr may, according to our initial hypothesis, potentially increase, in case this more spacious molecule would fit into the active center of mTyr (see below for results of the docking study).

In addition to the monooxygenase activity, mTyr also shows an oxidase activity (diphenolase activity). Via this pathway, catecholic substrates such as l-Dopa can be converted to dopaquinone by both mTyr present in the *met*-state (resting state; diphenolase activity) as well as the *oxy*-state (monophenolase and diphenolase activity),^28^ making it a high-level positive control for a rapid conversion without lag times. Therefore, l-Dopa was added as a reference substrate for enzyme activity tests to be compared to the here hypothesized artificial mTyr substrates DAT and DATT.

### 3.2 Docking Studies, MM-GBSA Calculations, and MD Simulations

To computationally investigate the hypothesis that DAT and DATT can be potential substrates for mTyr, we studied the interaction of DAT, DATT, and the natural substrate l-tyrosine with mTyr in dry-state docking studies using the crystal structure of mTyr (PDB ID: 2y9w).^44^ The docking revealed that all studied compounds were positioned in a similar manner within the binding pocket (Figure 2). As previously reported, the monophenol group of tyrosine forms a π-π stacking interaction with His263 and is oriented toward the CuB ion.^58^ In addition to this aromatic interaction, all compounds were found to form hydrogen bonds with the gate-keeper residue Val283.^59^ Notably, also the spacy DATT can enter the active center. DATT further engages with His244 and Glu322 through its second phenol moiety (Figure 2B). Interestingly, out of the 64 computationally generated conformers of DATT, the orientation of DATT in the substrate-enzyme complex was consistent, as illustrated in Figure 2B. This means that the second phenolic group of DATT, which formally is a DAT side chain, is unlikely to be converted but mainly serves to stabilize DATT in the active center. Thus, the docking studies revealed that DATT should not act as a bifunctional substrate.

**Figure 2:**
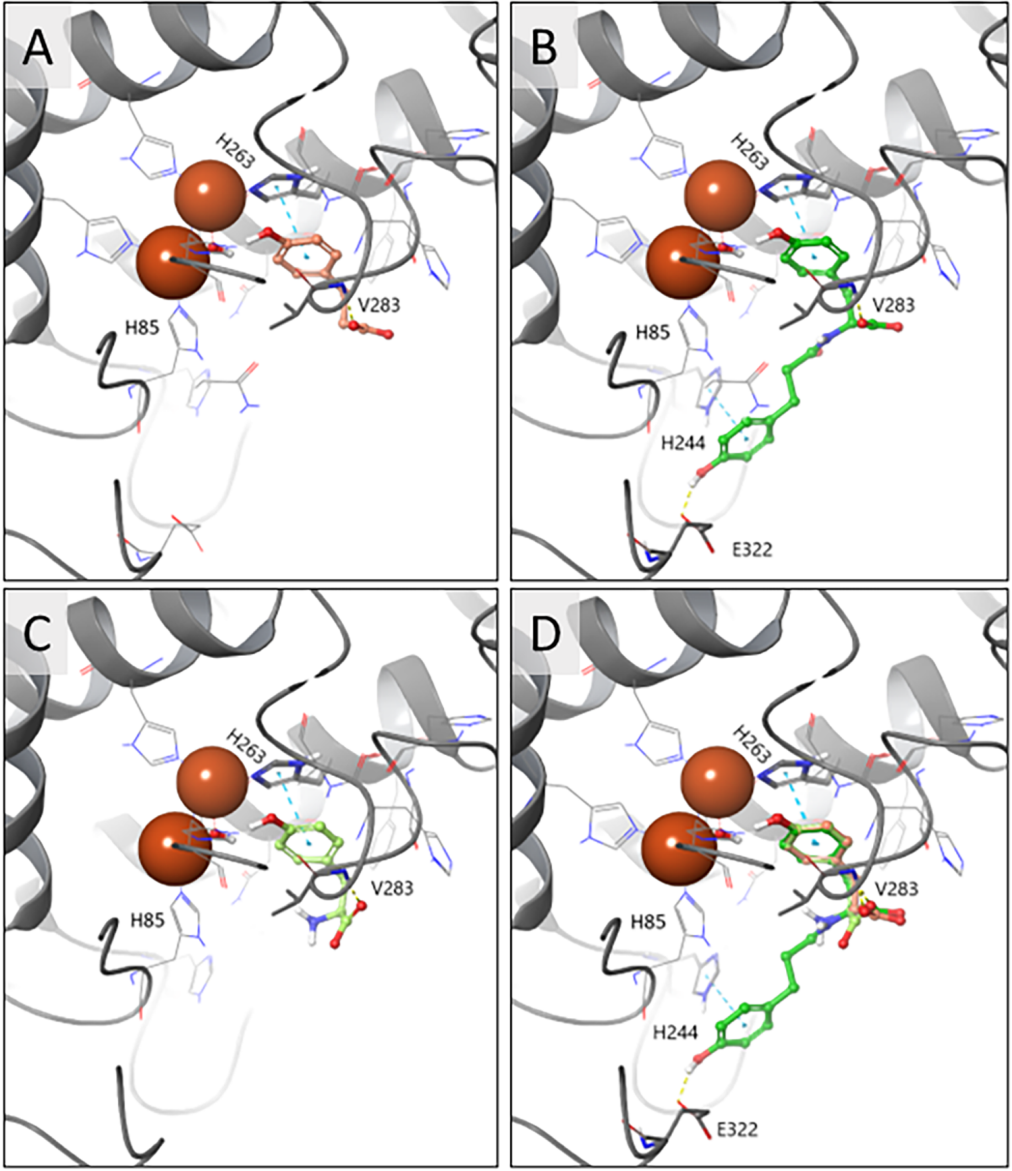
Docking poses at the mTyr binding site (PDB ID: 2Y9W) for (A) DAT (pink), (B) DATT (green), (C) tyrosine (lime), and (D) the overlay of all compounds. Hydrogen bonds are depicted as yellow dashed lines, and π-π stacking interactions are shown as cyan dashed lines.

In the next step, the binding affinities of DAT and DATT should be estimated in comparison to tyrosine, in addition to the previously determined docking scores. Binding energy values were obtained by calculating molecular mechanics with generalized Born and surface area solvation (MM-GBSA) (Table 1). This analysis of the enzyme–substrate complexes revealed that DAT exhibits a binding affinity similar to tyrosine, as can be seen from Δ*G*_Binding_ of ∼−30 kcal/mol for both DAT and tyrosine. These data support the hypothesis that artificial substrates such as DAT may access the active site of mTyr despite lacking the α-amino function. All compounds displayed favorable negative binding energy values, indicating stable interactions with the binding site. The major contributors to the binding energies were lipophilic (Δ*G*_Lipophilic_) and van der Waals (Δ*G*_vdW_) interactions, with a solvation penalty (Δ*G*_Solvation_) arising from the solvent-exposed pocket. DATT demonstrates a stronger binding interaction, as can be justified by additional H-bonding and π–π stacking through its second phenolic moiety.

**Table 1:** MM-GBSA Δ*G* Binding Energy Calculations and Docking Scores (kcal/mol)

ligand	docking score	Δ*G*_binding_	Δ*G*_lipophilic_	Δ*G*_Solvation_	Δ*G*_vdW_	Δ*G*_Coulomb(electrostatic)_	Δ*G*_covalent_	Δ*G*_H‑bond_	Δ*G*_packing_
tyrosine	–4.436	–30.94	–14.54	15.28	–23.88	–7.39	2.33	–0.26	–2.52
DAT	–4.504	–28.68	–15.11	15.98	–22.07	–8.08	3.62	–0.34	–2.69
DATT	–5.080	–47.77	–23.33	15.24	–38.66	–2.32	4.31	–0.62	–2.41

To validate the docking results, molecular dynamics (MD) simulations were performed in an aqueous environment for the complexes of mTyr with DAT, DATT, and tyrosine, respectively. These simulations should mimic the behavior of each complex in a physiological environment, where both the residues of the enzyme and the moieties of the substrates can move in the presence of water and ions. Based on RMSD-ligand and RMSD-protein values (Root Mean Square Deviation from reference structure at time point zero), DATT exhibits a substantial conformational flexibility (Figure 3A), which is much larger compared to DAT and tyrosine (Supporting Figure 1A,B). This flexibility may, despite the estimated favorable binding energies of DATT compared to the other substrates (see Table 1; molecular mechanics calculations in the dry-state docking), practically reduce the efficiency of binding and proper orientation of DATT within the active site of mTyr in the presence of water. In contrast, DAT and tyrosine formed more stable complexes with the enzyme, suggesting a potentially better compatibility for catalytic turnover. RMSF (Root Mean Square Fluctuation) analysis (Figure 3B) illustrated the movement of atoms of DATT during this MD simulation, which was high for the second phenolic group (carbons No. 1 to 8 and hydroxyl group No. 12., see labels in Figure 3B). Although DATT can engage in stable π–π interactions with His263 via its phenolic ring—similar to DAT and tyrosine—its additional phenolic moiety remains highly flexible and does not participate effectively in stabilizing interactions (Supporting Figure 1C).

**Figure 3:**
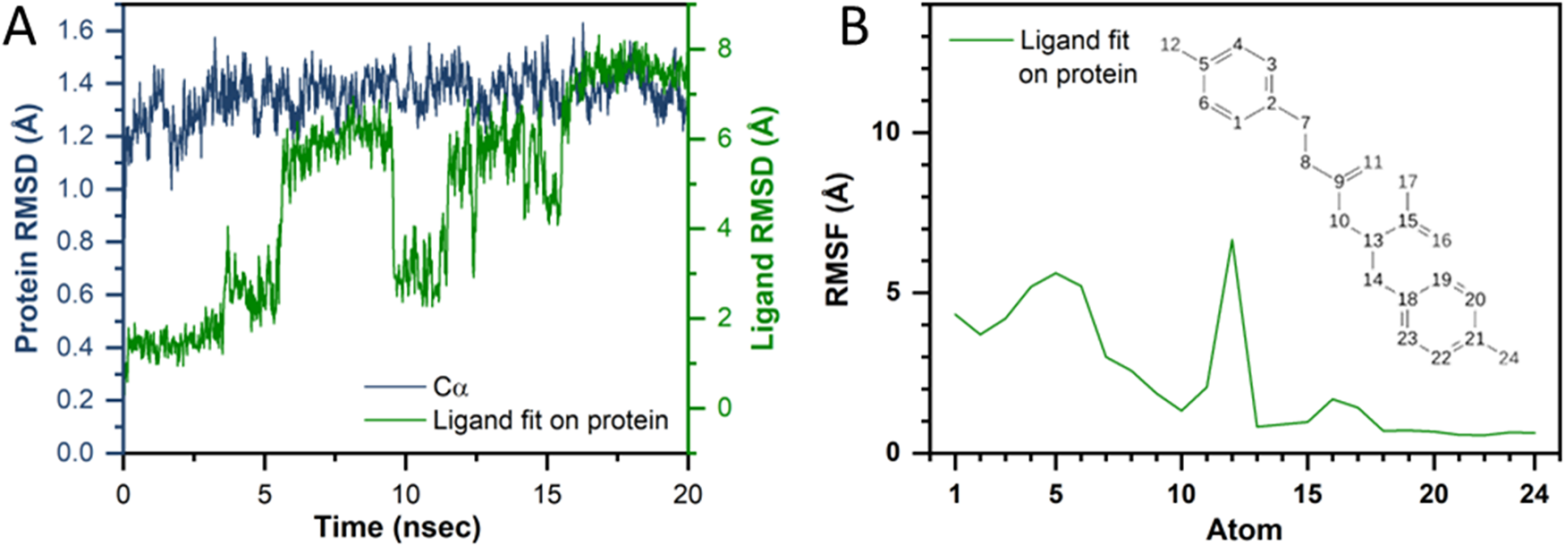
MD simulation analysis of mTyr with DATT. (A) Root Mean Square Deviation (RMSD) plots of the protein backbone (Cα) and DATT ligand over 20 ns simulations, indicating that the mTyr:DATT complex exhibits a reduced structural stability due to high ligand fluctuations. (B) Root Mean Square Fluctuation (RMSF) plot of ligand atoms showing that DATT displays substantial movement, particularly in the second phenolic ring, which may hinder stable interactions in the active site. Further information can be found in Supporting Figure 1.

To evaluate whether PEGylation alters substrate accessibility to the active site, additional docking studies were performed with OEG_6_-amide conjugates of DAT, DATT, and tyrosine (Supporting Figure 2) rather than the free substrates (as shown in Figure 2). Owing to their increased molecular size, only the terminal phenolic group of each OEG_6_ conjugate was able to approach the catalytic copper center, while the OEG backbone remained largely solvent-exposed outside the binding pocket. All PEGylated substrates could establish interactions with His85 in the CuA-proximal region, consistent with partially surface-exposed binding poses. PEGylated DATT showed the deepest penetration into the pocket; its second phenolic moiety was oriented toward the CuB region and retained a hydrogen-bond interaction with Val283. These results suggest that OEG_6_ conjugation does not prevent productive binding, but may reduce active-site accessibility and restrict optimal positioning for catalysis.

In the next step, MD simulation was employed to investigate whether OEG_6_ conjugation affects the dynamic behavior of the ligands when bound to the enzyme. For the OEG_6_-conjugated substrates, the protein backbone RMSD remained well-converged throughout the simulations, confirming that the bulky OEG_6_ scaffolds did not perturb the overall structure of mTyr. In contrast, the RMSD values (fit on the protein) of pegylated substrates were substantially higher for the OEG_6_ systems (Supporting Figure 3) compared to the free substrates (Supporting Figure 1), which can be attributed to the pronounced conformational flexibility of the OEG_6_ chains rather than to ligand dissociation. Molecular dynamics revealed distinct differences in how the three substrates interact with the His85 region of the catalytic site (Supporting Figure 4). OEG_6_-tyrosine remained in close proximity to His85 for the majority of the simulation, consistent with a productive positioning of the phenolic substrate. In comparison, OEG_6_-DAT exhibited a moderate increase in His85 distance over time but continued to intermittently resample near-productive geometries, indicating that the absence of the amino group in DAT introduces additional flexibility while still permitting access to the active site. In the case of OEG_6_-DATT, the phenolic headgroups remained close only in the early part of the trajectory, before undergoing larger conformational excursions. Overall, MD simulations reveal that OEG_6_ induces dynamic shielding of substrates to access mTyr, which slightly lowers the probability of catalytic alignment while still enabling productive binding events. Experimental studies of hydrogel synthesis from sOEG telechels coupled with artificial substrates showed that enzymatic conversion is possible (see Section 3.5).

Overall, the computational studies suggested the general suitability of the artificial substrates DAT and DATT for entering and being stabilized in the active center of mTyr. Based on the dry-state docking studies, binding energies were calculated to correspond to at least those of the natural substrate, tyrosine. Subsequent MD simulations in an aqueous environment suggested a good stability of the substrate-mTyr complexes for DAT and tyrosine, while the complex of mTyr with DATT was less stable in MD despite the more favorable binding energies in the docking study.

### 3.3 Evaluation of Specific Activities for Artificial and Natural Substrates

In the next part of this study, the suitability of the substrates for catalytic conversion by mTyr should be assessed. The predominant products of mTyr-based substrate oxidation are *o*-quinones, which subsequently react further to more complex structures. The formation of *o*-quinones can be assessed by the MBTH (3-methyl-2-benzothiazolinone hydrazine) assay with UV/vis spectrometric detection of pink addition products at 505 nm.^60^ The progress curves of the MBTH assay revealed differences for diphenolic (l-Dopa) and monophenolic substrates (l-tyrosine, DAT, DATT), the latter showing distinct lag times (Figure 4A). This observation for DAT and DATT was independent of the substrate concentration (data not shown), and correlated with earlier reports on oxygen consumption kinetics during conversion of phenols versus catechols by tyrosinase.^61,62^ The behavior can be explained by the oxidation state distribution of mTyr’s active site, which is present mostly (85%) in the *met* form (only diphenolase activity; allowing quick l-Dopa oxidation), while the *oxy* form of mTyr (monophenolase activity; converting tyrosine, DAT, and DATT) is generated only slowly, i.e., with a lag time, during catalytic activity by reaction of the *deoxy*-state of mTyr with molecular oxygen.^63,64^

**Figure 4:**
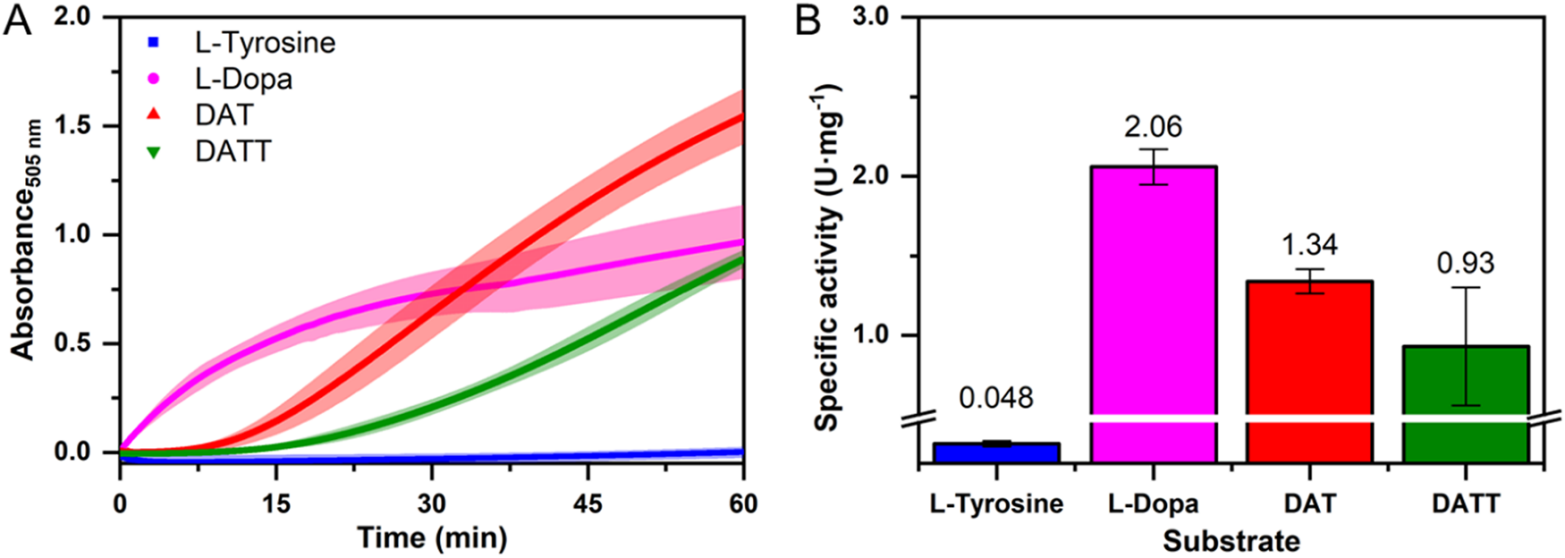
Conversion of natural and artificial substrates by mTyr, which are monophenolic (l-Tyrosine, DAT, DATT) or diphenolic structures (l-Dopa). (A) Progress curves of the MBTH assay. (B) Specific activity of mTyr based on the quantification of *o*-quinone generation with MBTH. Experiments with 1.1 nM mTyr and 1 mM substrate. Data presented as mean and SD of *n* = 3–4. In panel (A), the central lines correspond to the mean values of independent continuous measurements, while the shaded areas depict the SD.

Based on the slopes of the progress curves, the specific activities of mTyr for the natural and artificial substrates were determined. Remarkably, mTyr showed an extremely superior specific activity for DAT compared to the natural substrate l-tyrosine in O_2_-saturated buffer (Figure 4B). The specific activity for DATT was lower than for DAT, but still much higher than for l-tyrosine. The slower conversion of DATT and thus lower specific activity of mTyr for this substrate may be explained by the reduced structural stability in the active center (Figure 3). Additionally, a potential intra- or intermolecular clustering of phenols in DATT in aqueous solutions may occur,^65,66^ which conceptually may result in a lower ability to enter the active center. Therefore, DAT was selected as the preferred artificial substrate investigated in this study.

To evaluate the potential occurrence of steric shielding of phenolic substrates, another set of MD simulations with star-shaped sOEG_6_-substrate constructs was performed over the full 20 ns trajectories to calculate solvent-accessible surface areas (SASA). In DAT-sOEG_6_, all phenols remained highly solvent-exposed, with mean SASA values of approximately 70–75 Å^2^ per phenol, and only moderate fluctuations between individual branches (Supporting Figure 5). This indicates that sOEG_6_ does not bury the phenolic functionality in a compact conformation but keeps it readily accessible to the aqueous environment and, by extension, to the enzyme surface. In contrast, DATT-sOEG_6_ exhibited a slightly reduced overall mean SASA (∼65 Å^2^), consistent with increased steric crowding arising from the two phenolic rings per arm. Nevertheless, even in this case, no phenolic group became fully shielded from solvent, demonstrating that sOEG_6_ stars present multiple phenols in an accessible fashion capable of engaging the enzyme. Therefore, also DATT could be further included in this study.

Subsequently, the synthesis of the artificial substrates coupled to different types of linear (lOEG) and star-shaped (sOEG) materials was performed using EDC/NHS chemistry, resulting in telechelic molecules. FTIR spectrometry confirmed successful coupling by an increasing broad band with a maximum at 3340 cm^–1^ (secondary amines N–H stretching; phenol O–H stretching; hydrogen bonding) as well as broad bands at 1660 cm^–1^ (C=O stretching amid I) and 1550 cm^–1^ (N–H bending amid II) (Supporting Figure 6). An established ^1^H NMR method was employed to determine the degrees of DAT/DATT functionalization (*d*.*f*.) of sOEG,^67^ which reached values of 51–66 mol %.

### 3.4 Model Reactions to Elucidate Netpoint Chemistry and Functionality

In the second part of this study, the chemistry of netpoints and the predominant cross-linking routes were investigated by complementary techniques. We here concentrated on DAT only, as it was the most efficient and structurally least complex artificial substrate. Based on the literature on nonenzymatic catechol reactions,^31^ DAT-derived *o*-quinones may undergo spontaneous radical recombination, tautomerization, and reactions with nucleophiles such as amines, eventually leading toward larger cross-linked structures (Figure 5). Understanding the nature of netpoints of DAT-sOEG hydrogels is not only interesting on the level of fundamental science, but also a precondition for their potential medical use as discussed before. At the same time, investigating the nature of netpoints is highly challenging, given that mixtures of compounds are presumably formed that cannot be easily distinguished or separated. Attempts to isolate netpoint motifs from DAT-sOEG based hydrogel networks through basic hydrolysis (7.5 N NaOH, 70 °C, 4 d) of amide linkages between sOEG and the cross-linked end groups have not been conclusive. On the one hand, the materials were not fully degraded despite these harsh conditions. On the other hand, FTIR, ^1^H NMR, GPC, and MALDI-ToF MS analyses of extracted fractions indicated the presence of massive quantities of unexpected compounds with, e.g., aldehyde, carboxyl, hydroxyl, and/or lactone functionalities, possibly due to sOEG hydrolysis and oxidation.^68,69^ Therefore, this section of work focused on model reactions of DAT cross-linking using DAT derivatives that could be investigated without the need for an intermediate chemical treatment.

**Figure 5:**
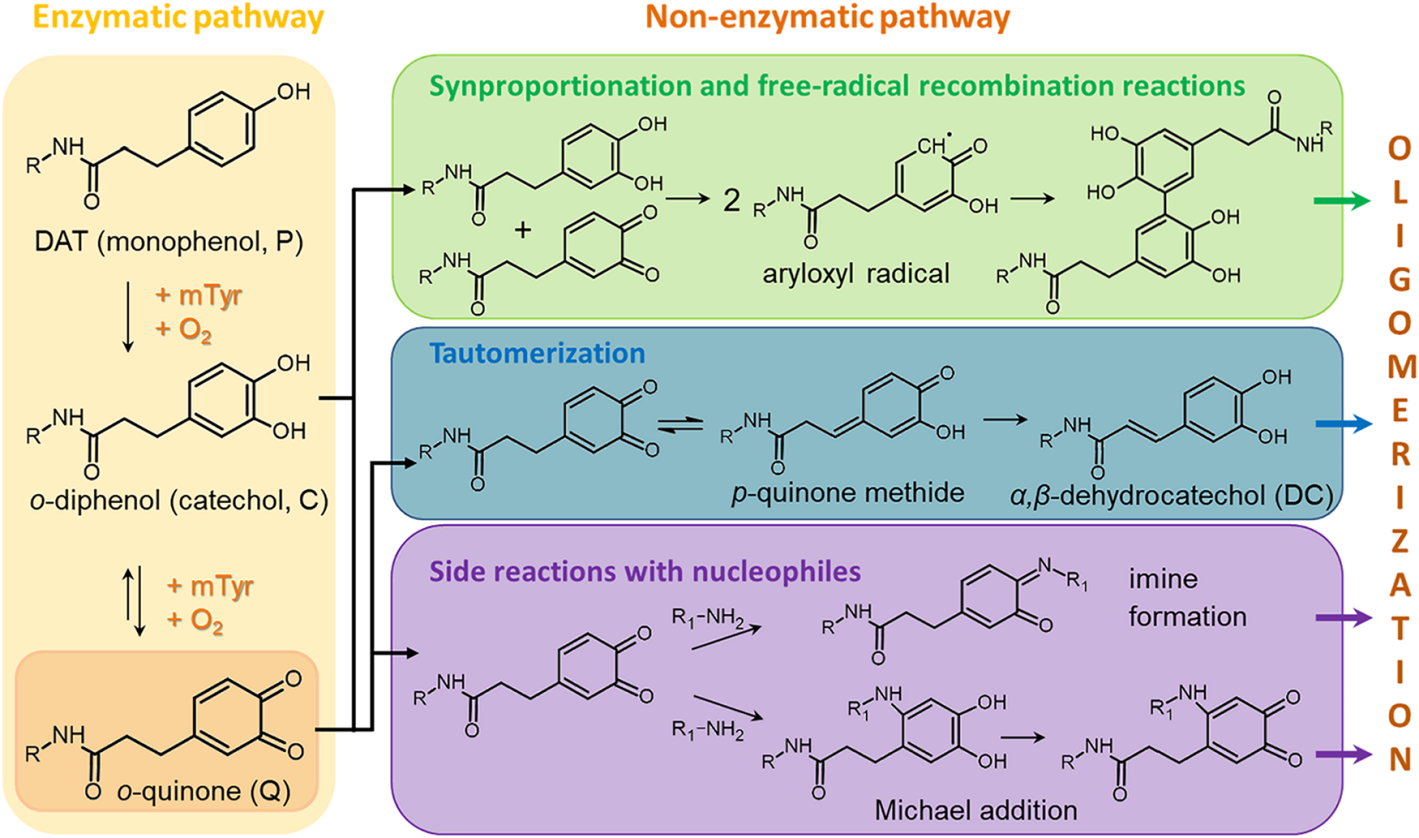
Possible reaction pathways of DAT for netpoint structure formation. Enzymatic reaction by mTyr leads to *o*-quinones. Subsequent nonenzymatic pathways can proceed through several mechanisms, in principle.

Depending on the analytical needs of the respective employed technique, DAT derivatives were used where the carboxyl function was modified with methanol (DAT methyl ester) or with different types of linear OEG bearing a methyl ether end group (lOEG_OMe_). In lOEG_OMe_, the methyl end group is not reactive, while the second terminus with an amine group was coupled to DAT. lOEG_oMe_ was used as a 10 kDa material (DAT-lOEG_OMe_ 10 kDa) corresponding to the standard molecular mass of sOEG. For spectroscopic techniques, linear 2 kDa oligomers were employed (DAT-lOEG_OMe_ 2 kDa) wherever dominant signals of longer OEG chains hamper the analysis of netpoint structures. For specific purposes in mass spectrometry, a highly monodisperse linear DAT-lmOEG_OMe_ 2 kDa was used.

In total, four subjects were addressed in the model reactions:

(i) A first task was to demonstrate the role of *o*-quinone formation from DAT in the overall reaction pathway. The above used MBTH assay confirmed the presence of *o*-quinones by Michael addition of Besthorn’s hydrazine to a pink product, thereby inhibiting any further reaction of the quinone. In principle, UV/vis spectrometry may also be able to detect both the formation and further conversion of *o*-quinones, if products show characteristic and distinguishable absorption bands. By using pure model phenols, catechols, quinones and coupled structures (without mTyr), characteristic differences in maximum absorption wavelength were observed: DAT at 276 nm, catechol at ∼280 nm, and alkyl-chain substituted *o*-quinones at ∼400 nm (Supporting Figure 7). Shoulders along the main peaks such as at ∼310 nm are indicative of unsaturated alkyl chains substituents of quinones. Literature also reported absorption peaks at 410 nm for *o*-diphenol dimers^70^ and 420 nm for peptide-substituted *o*-diphenol dimers,^71^ the latter being confirmed by mass spectrometry.
  DAT-lOEG_OMe_ 10 kDa showed a relatively sharp UV absorption band at 274 nm (time point 0 h) when explored either at diluted conditions (6.25 mg·mL^–1^; Figure 6A) or at a polymer concentration close to standard concentrations used for hydrogel formation (50 mg·mL^–1^; Figure 6B). Upon enzymatic reaction, a slight bathochromic shift of the main peak was observed within 15 min after starting the reaction, with a peak broadening to form a shoulder at 320 nm that further expanded to higher wavelengths over 24 h. A further peak at 405 nm (best visible in diluted conditions, Figure 6A) formed within the first minutes after starting the reaction and declined with time. In summary, these data confirmed the formation of *o*-quinone intermediates (405 nm absorption band) and their consumption by further nonenzymatic reaction, but also suggest the occurrence of *o*-diphenols (peak maximum shift to 280 nm) and α,β-unsaturated DAT derivatives (shoulder at 320 nm).
  The oxidation of the DAT groups of DAT-lOEG_OMe_ 2 kDa was also confirmed by FTIR spectroscopy (samples freeze-dried after reaction) based on the disappearance of signals for aromatic ring skeletal vibrations (1780 cm^–1^) and the aromatic C=C stretch (1515 cm^–1^) (Figure 6C). This was further supported by decreased intensities of bands at 1180 cm^–1^ (assumed C–O stretching vibrations of phenolic O–H groups) and 705 cm^–1^ (assumed phenolic O–H out-of-plane bending vibration). Despite the long reaction time (7 d) as chosen to reach end-point conditions, there was also an indication of some remaining *o*-quinones, potentially part of larger cross-linked structures. This conclusion was based on a new band at 1200 cm^–1^ assigned as C–C stretching adjacent to ketone groups. HPLC-ESI-MS spectrometry of DAT-lmOEG_OMe_ 2 kDa showed the absence of the chromatographic peak and ion traces of the analyte after 24 h of reaction with mTyr (Supporting Figure 8), demonstrating that the conversion of DAT by mTyr is quantitative.
(ii) As a second subject of the model reactions, the number of end groups linked in netpoints should be evaluated by the fold increase of molecular weights of the model compounds. In MALDI-MS analysis, the oligomeric reaction products of DAT-lOEG_OMe_ 10 kDa after conversion by mTyr could not be properly ionized and analyzed. Therefore, as an alternative model component with a minimum size of the substituent at the DAT carboxyl function, DAT methyl ester was employed. The analysis of product precipitates formed upon incubation of DAT methyl ester with mTyr resulted in compounds of *m*/*z* up to 2250, corresponding to undecamers (Figure 7A). This suggests that very large netpoint structures can be expected in principle. However, for polymer-coupled DAT as substrates, additional aspects may come into play. On the one hand, steric restrictions and the higher viscosity of polymer solutions may reduce the required substrate and oxygen diffusion to mTyr and subsequently the diffusion of converted moieties to their reaction partners, potentially leading to fewer chains linked per netpoint. On the other hand, while not observed in the simulation in Supporting Figure 5, a clustering of DAT units in an aqueous environment^72^ may promote larger netpoints. Overall, this analysis showed that multimer netpoints can be formed from polymer-coupled DAT.
(iii) The chemical structure of the netpoint motifs should be evaluated as a third topic of the model reactions. As indicated in Figure 5, *o*-quinones can enter into nonenzymatic reactions, such as via synproportionation with diphenols to form aryloxyl radicals, tautomerization of *o*-quinones (*Q*), eventually leading to α,β-dehydrocatechol (*DC*) as new reaction partners, and/or reaction with nucleophiles, which includes but is not limited to amines. While the polymerization of *DC* derivatives is mechanistically not understood in detail,^31^ it is obvious that the availability of both *Q* and catechols (*C*) in the reaction mixture (see Figure 5, left box and arrows to upper left box) suggests the occurrence of aryloxyl-based recombination reactions as the commonly accepted main pathway. Apparently, it was not possible to conclude from product masses detected in MALDI-ToF-MS (Figure 7A) on the relative positioning of the linkage between the constituents, the order of coupled precursors, and the respective pathways of cross-linking. Despite starting from DAT methyl ester only, a wide spectrum of structural motifs could be derived thereof (Figure 7B), which may participate in the nonenzymatic oligomerization reactions. It should be noted that some motifs are structural isomers, which have identical masses and could not be distinguished (e.g., *Q* and *DC*). Besides direct C–C coupling, ether-type bridges are also possible (Figure 7C).^73^
  Due to decreasing signal intensity and combinational complexity for larger multimers, the peak assignment was attempted for trimers to heptamers (Figure 7D). For each newly formed bond during the oligomerization, the product mass is reduced by two protons compared to the masses of the monomeric motifs, while ionization during analysis leads to an increase of the observed masses depending on the respective adducts (typically [M + Na]^+^). The data interpretation suggests that the multimers predominantly contained *C* and hydroxycatechol (*HC*) structures, but also some *Q* (or *DC*, its product of tautomerization) motifs, as well as catecholic DAT derivatives with a free acid (*CA*). The presence of *Q* motifs in multimers suggests that *Q* is not quantitatively subject to synproportionation to *Q* ^·^, but also other reaction pathways such as reaction with nucleophiles can be involved (see Figure 5). For *DC*, a number of coupling reactions not only at the ring structure but also at the α,β double bond of the alkyl chain may occur, including β-O or β-β*′* bonds, as suggested by structural similarity to pathways in lignol coupling.^73^ For most signals, some alternative assignments were also possible (Supporting Table 1). Some of these alternative assignments include monophenolic DAT methyl ester (*P*), i.e., the substrate without enzymatic oxidation, to be integrated in multimers. This might be possible due to the relatively high initial concentration of *P* in the reaction mixture and the rate-limited character of enzymatic phenol hydroxylation. Such incorporation of *P* suggests, at least in principle, that also DAT end groups of polymeric telechels that are not activated by mTyr might theoretically be incorporated in polymer networks.
  The position of cross-links in pure substances can often be revealed by ^1^H NMR. Here, however, mixtures of substances were present and reaction products were only partially soluble. Still, an attempt was made to evaluate the information extractable by ^1^H NMR from these complex samples. The most pronounced changes of signals after DAT conversion are expected for its aromatic protons (doublets at 6.75 and 7.1 ppm in D_2_O), since ring adducts are supposed to be predominant for netpoint formation. From a software-assisted prediction of ^1^H NMR spectra, it was obvious that chemical shifts of adjacent protons for different positions of C–C coupled *C* motifs, e.g., in 5*′*-5*′* versus 6*′*-6*′* position, are marginal, while, e.g., cross-linked structures containing *C* and *Q* might be distinguishable by signals of *Q* at ∼ 6.5 ppm (Supporting Table 2). Then, experimental data were collected for DAT methyl ester and DAT-lOEG_OMe_ 2 kDa incubated with mTyr, followed by aliquot collection at different time points, shock freezing, freeze-drying, and later dissolution (in some cases in MeOD-d4 due to solubility issues; peak shifts to lower δ by 0.05–0.1 ppm expected relative to D_2_O). For DAT methyl ester derived samples, the sharp signals of the aromatic protons of the phenol moiety at 6.7 and 7.0 ppm converted into various sharp peaks as determined after 0.25 and 1 d, followed by complete transition into a very broad overlapping signal after completing of the reaction after 3 d as characteristic for very heterogeneous mixtures (Supporting Figure 9A). The spectra indicate the formation of *o*-quinones (6.55 ppm) and catechols (6.6–6.7 ppm, partially overlapping with signal of phenolic protons in 2′/6′ position at 6.7 ppm) as intermediates within the first hours of reaction, with remaining signals in this region also after completed reaction. Interestingly, new signals at 6.2 to 6.7 ppm were observed during analysis after 1 d of reaction onset, which might be indicative of aromatic protons next to newly formed C–C linkages between two ring structures, such as a 5′-5′ coupling. For DAT-lOEG_OMe_ 2 kDa samples, only minor changes of the spectrum were observed after 1 d; however, the sharp signals of the phenolic protons had disappeared after 7 d giving rise to a very broad signal probably associated with various coupled compounds (Supporting Figure 9B). Also changes in the aliphatic region (2.3–3 ppm) were detected, which is in line with the simulation that has suggested slightly different chemical shifts of aliphatic protons for different oxidation states of the adjacent ring system. The differences in reaction kinetics of DAT-lOEG_OMe_ 2 kDa and DAT methyl ester correspond to literature reports on 1*′* substituted catechols, where increasing length and hydrophilicity of small molecule substituent reduced the oxidation rates by mTyr.^74^
(iv) The fourth subject for model reaction addresses the participation of amine groups in cross-linking, which are present in sOEG-amine (Figure 1), the educt used to synthesize DAT-sOEG telechelics. As the DAT functionalization of sOEG is not quantitative, some remaining amine groups may be supportive to network formation by acting as nucleophiles (Figure 5). Literature suggests the reaction of amines with *o*-quinones leading to catechol derivatives as Michael addition products^75,76^ that may be further oxidized to quinone derivatives.^31,77^ Equimolar mixtures of DAT-lmOEG_OMe_ 2 kDa and NH_2_-lmOEG_OMe_ 2 kDa were incubated with mTyr and analyzed by MALDI-ToF MS (Figure 8). No signals linked to Schiff’s base (imine) formation between the reactants were detectable under the given analytical conditions. Instead, signals of Michael addition products of NH_2_-lmOEG_OMe_ and DAT-lmOEG_OMe_ derived quinone (*m*/*z* 4472.125) were observed in the dimer region of the spectrum (theoretical mass [M + Na]^+^ 4471.6054). This suggests that the reaction pathway with nucleophiles is practically occurring in the investigated material system. Therefore, any arms of sOEG-amine that lack a DAT end group will still be active as reaction partners for network formation. Based on the signal decay in the monomer region, this investigation also allowed to conclude on the relative contribution of the coupling reaction with amines relative to other pathways. Starting from an equimolar mixture of amine- and DAT-bearing lmOEG_oMe_, only signals for noncoupled NH_2_-lmOEG_OMe_ remained visible as the dominating signals of the MS spectrum. In contrast, DAT-lmOEG_OMe_, as well as catechol or quinone derivatives, were not detectable and thus fully consumed by competitive reactions dominating over Michael additions with amines. Thus, the reaction of *o*-quinones with amines has apparently be slower than radical recombination.^77^ The nonquantitative incorporation of NH_2_-lmOEG_OMe_ may further be explained by the selected reaction pH at physiological conditions, where the amine moieties are mostly protonated (p*K*_a_ > 9 expected; reference p*K*_a_ of 2-aminoethanol = 9.5 ^78^) and thus their nucleophilicity is low.

**Figure 6:**
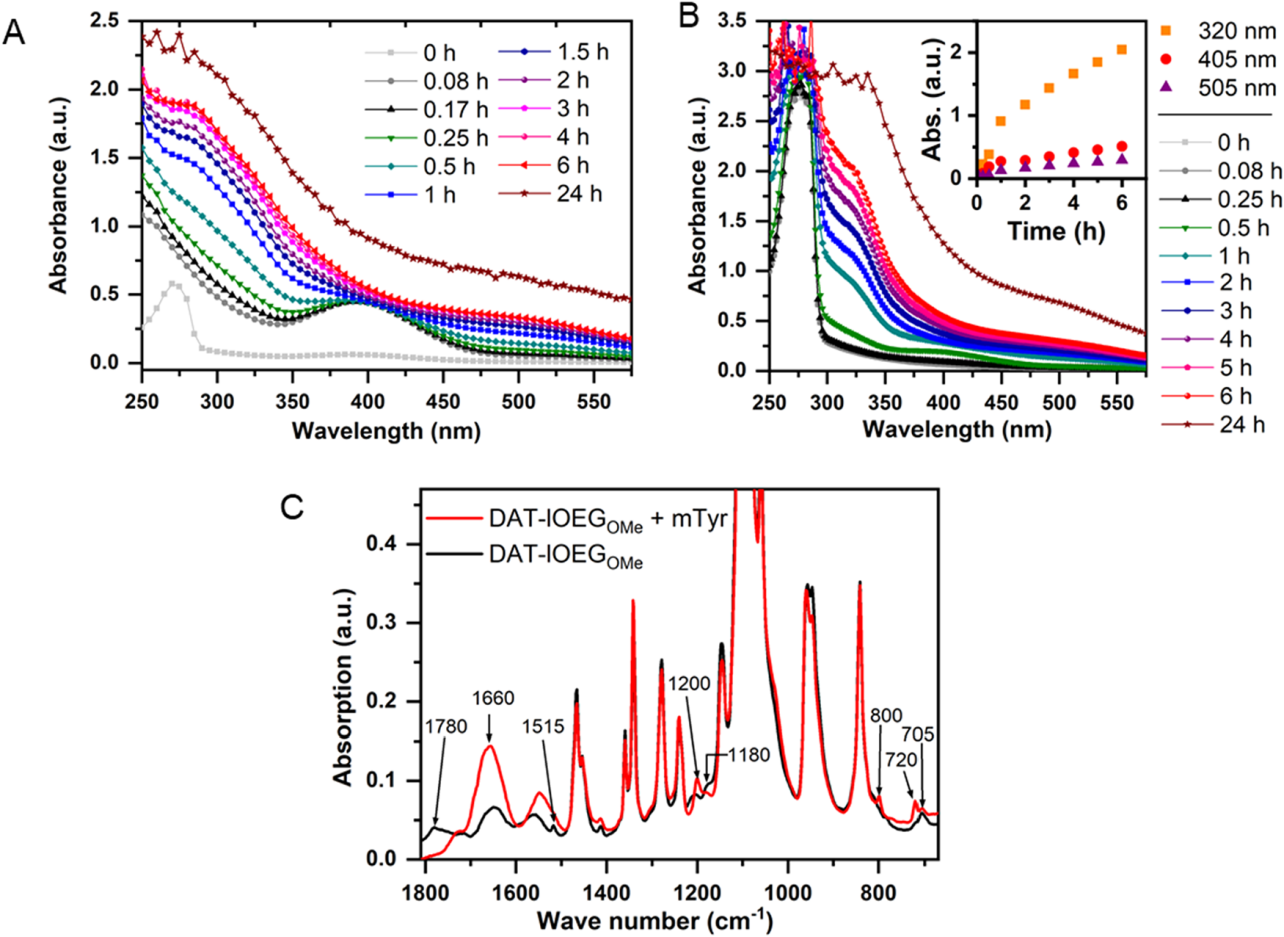
Model reaction of DAT-lOEG_OMe_ with mTyr monitoring conversion and reaction intermediates. (A-B) UV/vis spectrometry of DAT-lOEG_OMe_ 10 kDa (d.f. 62 mol %) at different polymer/mTyr ratios: (A) 6.25 mg·mL^–1^ polymer, 500 U·mL^–1^ mTyr, (B) 50 mg·mL^–1^ polymer, 10 U·mL^–1^ mTyr (diluted prior to UV measurement). The inset shows the change of absorbance at selected wavelengths plotted against time. (C) FTIR analyses of freeze-dried DAT-lOEG_OMe_ 2 kDa (d.f. Thirteen mol %) before and after reaction with mTyr (70 mg·mL^–1^ polymer, 2000 U·mL^–1^ mTyr, 7 d reaction time).

**Figure 7:**
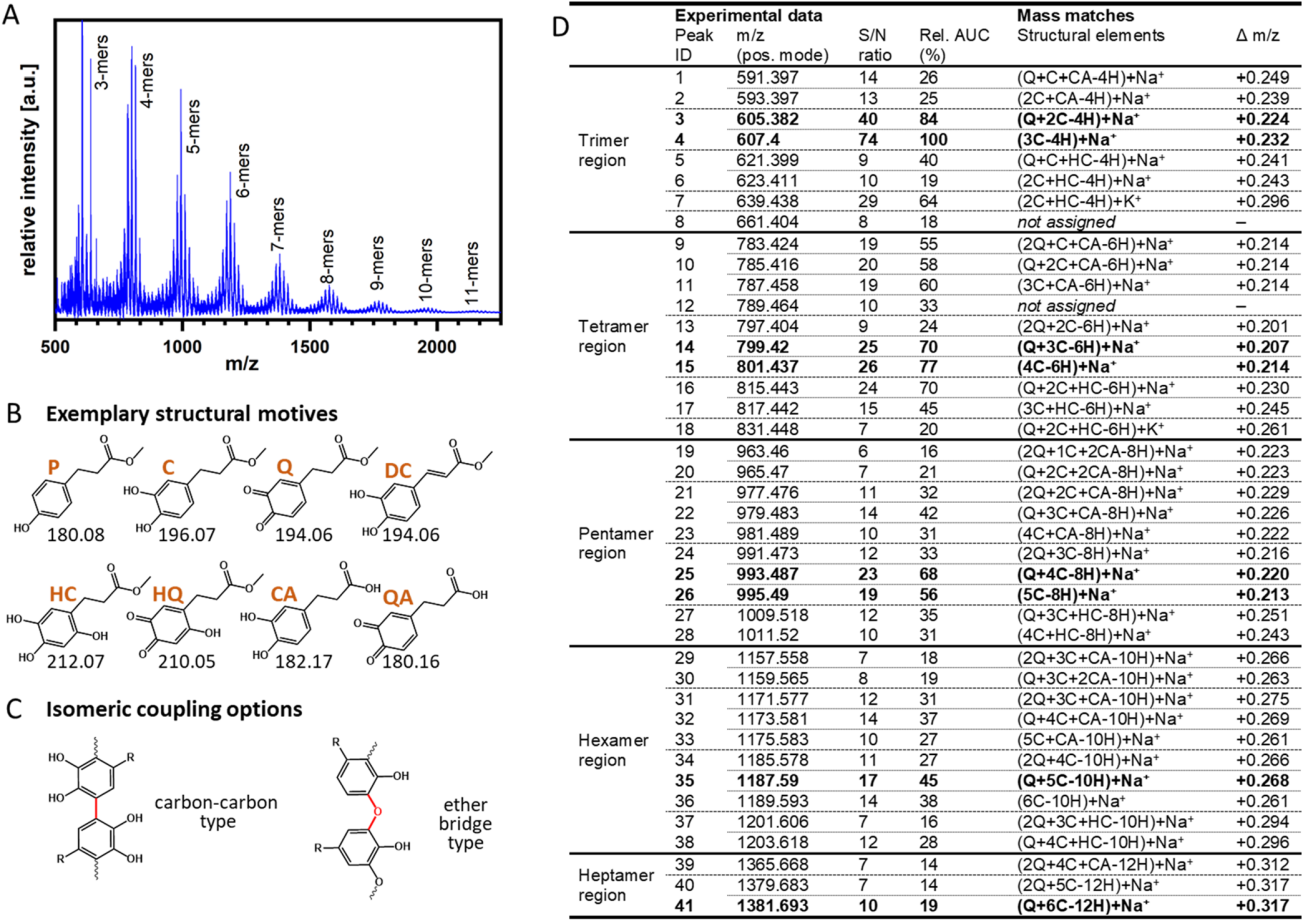
Analysis of netpoint functionality and netpoint structures using DAT methyl ester as a model substrate for mTyr. (A) MALDI-ToF MS spectrum. (B) Exemplary structures that may be derived from DAT methyl ester, as well as their monoisotopic masses. *P*: phenol (DAT ester); *C*: catechol; *Q*: *o*-quinone; *DC*: α,β-dehydrocatechol; *HC*: hydroxycatechol; *HQ*: hydroxy *o*-quinone; *CA*: catechol derivative with free acid; *QA*: *o*-quinone derivative with free acid. *DC* is obtained by tautomerization of *Q*. *HC* is the product of the reaction of *Q* with water. *HQ* may be obtained by oxidation reaction of *Q*. *CA* and *QA* are derivatives of *C* and *Q* with a free carboxylic acid function after methyl ester hydrolysis. In principle, DAT-derived multimers can contain all structural motifs as depicted above. (C) Coupling can proceed in various positions and through different types of bonds, such as carbon–carbon linkages and ether bridge-type links to 2*′*, 5*′*, or 6*′* positions of the next multimer unit. Two exemplary isomeric structures are shown. (D) Interpretation of multimer signals. The product mass (*m*/*z*) of multimers lacks an equivalent of two protons for each newly formed bond (relative to the sum of the masses of the individual motifs). Main signals of the spectra are printed in bold. For alternative assignments and more explanation, see Supporting Table 1.

**Figure 8:**
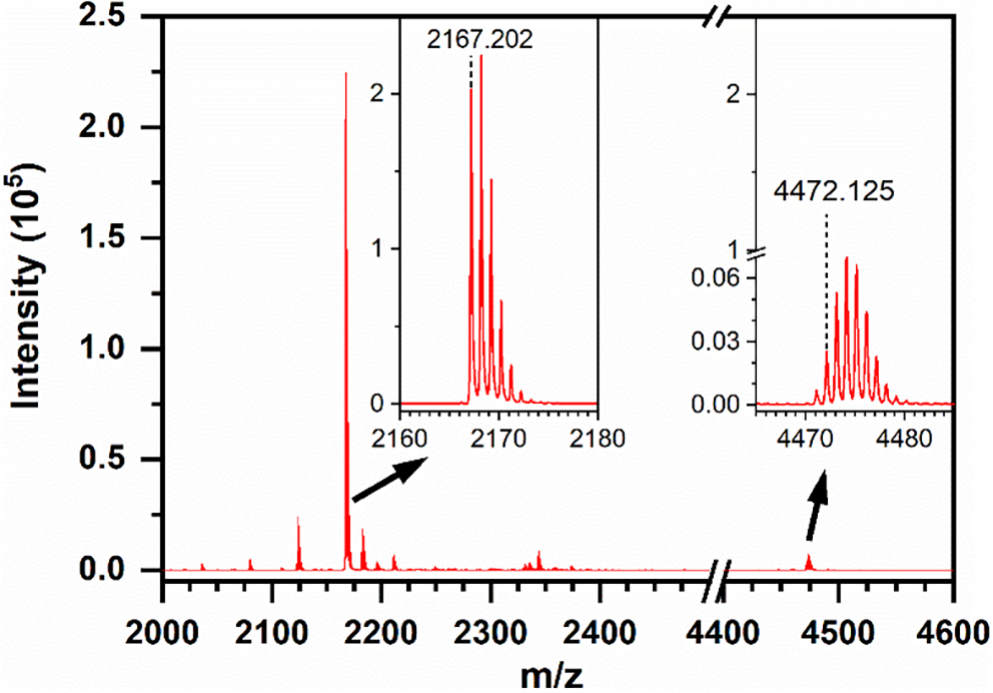
Model reaction investigating the participation of amine groups as a supportive reaction for network formation (MALDI-ToF MS spectra). DAT-lmOEG_OMe_ 2 kDa and NH_2_-lmOEG_OMe_ 2 kDa mixtures (1:1 molar ratio) were incubated with mTyr, and the reaction mixture was analyzed after 24 h (total oligomer conc. 5 wt %; O_2_ saturated PBS buffer; mTyr 1000 U·mL^–1^). Inserts show peaks of Na^+^ adducts of remaining NH_2_-lmOEG_OMe_ ([M + Na]^+^ *m*/*z* 2167.202; Δ*m*/*z* + 0.0877) as well as the Michael addition products of NH_2_-lmOEG_OMe_ and DAT-lmOEG_OMe_ derived quinone ([M + Na]^+^ *m*/*z* 4472.125; Δ*m*/*z* + 0.52). No relevant signals for remaining DAT-lmOEG_OMe_ 2 kDa ([M + Na]^+^ *m*/*z* 2315.342) or noncoupled DAT-OEG derivatives were detected (Quinone-OEG [M + Na]^+^ 2329.32; Catechol-OEG [M + Na]^+^ 2331.33; DAT-OEG [M + Na]^+^ 2315.342; Hydroxycatechol-OEG [M + Na]^+^ 2347.332).

Overall, the set of model reactions revealed not only intermediates and reaction products formed from DAT following the enzymatic oxidation, but also showed the nature of supportive reactions involved in forming sOEG-based hydrogel networks.

### 3.5 Enzymatic Hydrogel Synthesis and Characterization of Structure and Properties

The third part of this study should demonstrate the feasibility of mTyr to build functional hydrogel materials from DAT/DATT-sOEG. Structure–function relationships should be evaluated upon altering the substrate type (DAT/DATT), the enzyme concentration, and the molar mass of the sOEG units, as associated with the relative content of end groups at a fixed oligomer concentration of 5 wt % (Table 2). In the first step, the ability for gel formation by, e.g., 100 U·mL^–1^ mTyr was evaluated by a tube tilting assay with visual examination after up to 24 h. For DAT/DATT functionalized sOEG of 5 kDa to 20 kDa, gelation was observed for all materials except DATT-sOEG 5 kDa, which showed coloration but no homogeneous gelation (excluded from further analysis).

**Table 2:**
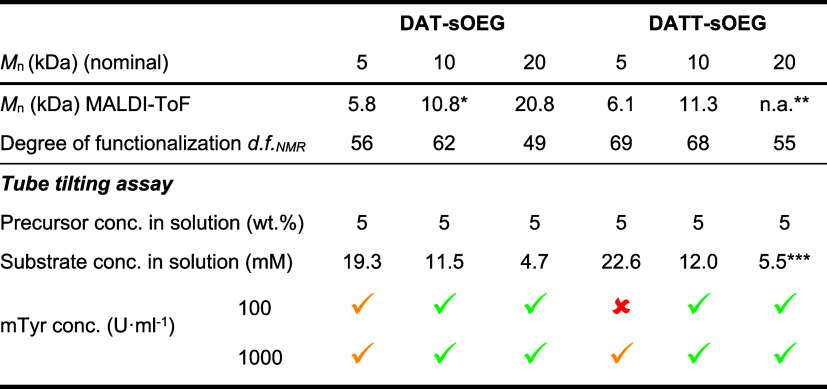
Tube Tilting Assay for Screening of Hydrogel Gelation Pattern^a^ ^,^ [Table-fn t2fn2]

a Presented data include properties of DAT/DATT functionalized sOEG material batches used for screening of gelation as well as the appearance of samples after exposure to 100 or 1000 U·mL^–1^ mTyr (room temperature; 5 wt.% precursor in oxygen-saturated PBS pH 7.4).

b
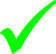
Gelation within 6 h; 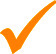
Partial or inconsistent gelation within 16 h; 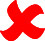 No reproducible gelation within 24 h; * large quantities of smaller molar mass OEG detected as contamination; ** not analyzed; data of comparable synthesis indicate Mn of 21.2 kDa; *** Nominal Mn used for calulation.

To better understand the gelation kinetics depending on mTyr concentration (100–1000 U·mL^–1^), the alteration of complex viscosity η* during the enzyme-induced reaction was monitored by oscillatory rheology, where a steep increase of η* marked the gel time *t*_gel_ (Supporting Figure 10). Generally, gelation occurred within a few minutes up to several ours in a very systematic pattern depending on enzyme concentration and the molecular weights of the telechelics. Similar time scales of gelation were observed for DAT- and DATT-functionalized telechelics at a given enzyme concentration, with some minor trends toward faster cross-linking when DAT was the substrate. Interestingly, cross-linking was in all cases faster for telechelics with higher molar masses (see Supporting Figure 10A–C as well as Supporting Figure 10D,E), despite the substrate concentration being relatively lower with increasing molar masses (see Table 2 for correlation of molar masses and substrate concentration). The positive effect of higher molar masses may be linked to increasing chain flexibility, increasing radius of gyration, and/or stronger contributions of chain entanglements to network fixation. The rheological data confirmed the results of the tube tilting assay and are also in line with the ranking of artificial substrate conversion by mTyr (see Figure 4). Two mTyr concentrations, 100 and 500 U·mL^–1^, were selected for subsequent experiments as they represent slow and faster gelling conditions.

Differences in gelation kinetics do not necessarily mean that product properties have to be different. In fact, in many applications, such as for technical glues, it is advantageous to have fast and slow curing versions that reach similar final properties, such as mechanical properties. Therefore, the final hydrogel properties were assessed after gelation overnight, followed by drying for ≥3 d. The investigated characteristics included the gel content *G* (describing the amount of telechelics successfully incorporated in the network material), the degree of volumetric swelling *Q* (being a measure of the network’s cross-linking density), and the storage modulus *G*′ (indicating the elastic properties of the network material), all being linked to the molecular structure of the hydrogel. It was very interesting to see that despite the different cross-linking kinetics, 10 kDa and 20 kDa telechelics with both DAT and DATT as enzymatic substrates had similarly high *G* around 80 wt % (Figure 9A). Higher quantities of extractables, i.e., predominantly originating from nonincorporated telechelics, were observed only for shorter chain DAT-sOEG 5 kDa at low mTyr concentration. This is in line with the previous observations of poor gel formation in the tube tilting assay (Table 2) and the investigation of cross-linking kinetics (Supporting Figure 10). For short-chain sOEG 5 kDa, mobility restrictions of DAT might have hindered effective conversion by the sparsely available enzyme.

**Figure 9:**
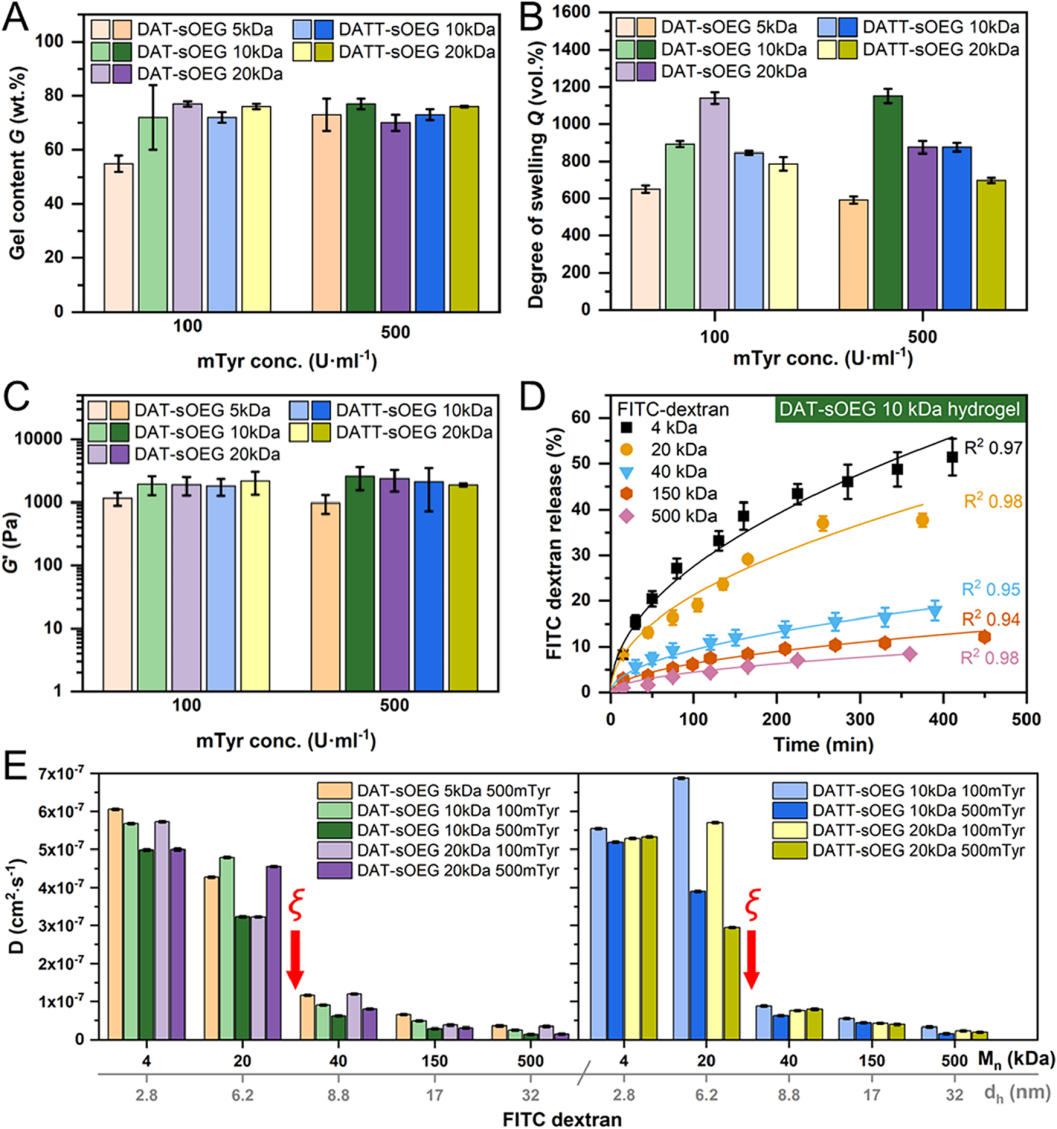
Characterization of hydrogel properties as synthesized by variation of precursor molar mass (5, 10, 20 kDa), type of substrate (DAT, DATT), and enzyme concentration (100, 500 U·mL^–1^). (A) Gel content *G* after aqueous extraction for 24 h (*n* = 3). (B) Degree of swelling *Q* in PBS buffer pH 7.4 at 37 °C for 24 h (*n* = 6). (C) Storage modulus *G′* from time-sweep analysis by oscillatory rheology conducted with preformed and previously extracted gel samples at 37 °C (*n* ≥ 3). (D, E) Diffusion experiments with FITC-dextran of different molar masses (4, 20, 40, 150, 500 kDa) incorporated in thin flat hydrogel samples, which were covered with PBS buffer pH 7.4 at 37 °C (*n* = 4). (D) Exemplary release curves of FITC-dextran from DAT-sOEG 10 kDa derived hydrogels. (E) Diffusion coefficients of FITC-dextran in the hydrogels (as calculated by fitting release curves with a diffusion model^53^) and estimation of the functional average mesh size of the hydrogels based on an approximation of the hydrodynamic diameter of the FITC-dextrans. Data presented as mean and SD.

The next analysis, which was the investigation of *Q*, again revealed a clear effect of precursor chain length for 5 kDa with the expected lower degree of swelling. Additionally, an effect of enzyme concentration was observed at least for DAT-sOEG 10 and 20 kDa (Figure 9B), which formed tighter networks with lower swelling at higher (500 U·mL^–1^) mTyr concentration. The availability of more enzyme molecules might have resulted in more parallel early phase cross-linking, resulting in a more entangled network structure. DATT, in contrast, appears to reach maximum cross-link density under the conditions tested already at lower enzyme concentration (100 U·mL^–1^), potentially due to the availability of two phenol moieties per end group and thus more hypothetical sites that can be enzymatically activated for subsequent cross-linking reactions.

In addition to the previous investigation of cross-linking kinetics by rheology, preformed hydrogels after extraction of solubles were also studied by rheology. This investigation showed linear curves of *G*′ during frequency sweeps over several orders of magnitude, which is indicative of an elastic, soft, solid-like material with a good resistance to mechanical stress.^79^ In the linear viscoelastic region, *G′* adapted values of 1 to 3 kPa for all samples (Figure 9C). The obtained similar mechanical properties suggest a strong contribution of DAT/DATT independent features to hydrogel mechanics, e.g., through entanglements of sOEG chains and netpoints provided by the central sOEG carbon.

The average mesh size ξ of hydrogels, i.e., the average linear distance between two adjacent cross-links, is an important characteristic of their molecular structure. It affects the mobility of molecules within the material as well as the diffusion of solutes into and out of the hydrogel.^80^ With some exceptions of relatively homogeneous architectures obtained under certain conditions,^18^ molecular architectures in systems as studied here are commonly inhomogeneous by design. This is because of a potential polydispersity of the networks’ functional units, sOEG, and differences in the length of the respective arms of sOEG, different types of netpoints and number of coupled OEG chains (see model reactions), the clustering of hydrophobic moieties, occurrence of chain entanglements and dangling chains, etc. Thus, it should be noted that average mesh sizes do not represent definite (sharp) exclusion values, but indicate that changes in relative diffusivity may occur at sizes clearly above and below this value. From *G′* data of previously extracted DAT/DATT-sOEG hydrogels, ξ values in the range of 13 to 16 nm have been calculated (Supporting Table 3) when applying rubber elastic theory to this system.^81^ As hydrogels are swollen in a solvent that reduces molecular interaction between polymer chains, thus allowing for high chain mobility, the ξ values calculated from rheological data do not necessarily correlate with a functional ξ for diffusion control.

Therefore, it was also intended to determine functional ξ values based on the diffusivity of molecules with different hydrodynamic diameters. As easily detectable probes,^82,83^ FITC-labeled dextranes of 4 to 500 kDa were incorporated in the DAT-sOEG and DATT-sOEG hydrogels during their synthesis as thin films in microtiter plates. The samples had a plane geometry, a defined thickness, and a homogeneous FITC-dextran distribution. They were covered with medium to study the FITC-dextran diffusion out of the samples. From the release curves, as exemplarily illustrated in Figure 9D, a scaling effect was obvious, showing restricted diffusion in correlation with FITC-dextran size.

Due to the selected test geometry, a concentration gradient was built only toward the surface of the sample, and the Fickian diffusion law was applicable,^84^ allowing for the determination of diffusion coefficients *D*_g_ of FITC-dextrans in the gel after curve fitting by a power law.^53^ Correlation coefficients of 0.93 to 0.99 for the fitting of most experimental curves suggested that a diffusion-controlled transport was applicable, with a low impact of physical interaction between the hydrogel network material and the FITC-dextrans. By trend, *D*_g_ was only slightly reduced by higher mTyr concentration for network synthesis (Figure 9E). However, a drop of *D*_g_ was observed when changing from 20 to 40 kDa FITC-dextran. In a simplified model, dextrans may be handled as solid spheres to calculate rough estimates of their hydrodynamic diameters *d*_h_ by the Stokes–Einstein equation (see second *x*-axis in Figure 9E), which are values similar to empirical equations or experimental data of *d*_h_ of dextrans in literature.^85,86^ This suggests that all hydrogels had a functional ξ in the range of FITC dextran 40 kDa, i.e., a *d*_h_ of ∼9 nm. Still, it should be noted that solvents that include dissolved (buffer) ions affect *d*_h_ by changes in chain coiling. Additionally, the available dextrans are not monodisperse (polydispersity of 1.4 to 2.0 according to the manufacturer’s certificate). Therefore, some deviations in this estimation of *d*_h_ should be accepted. Interestingly, also FITC dextran with a molar mass as high as 500 kDa was not quantitatively retained in the material, but showed slow diffusion (Figure 9E). This emphasizes that real-life hydrogel networks do not build a quantitative diffusion barrier for molecules with *d*_h_ far above their ξ, which—importantly—is an average mesh size and is typically subject to local defects.

In the next step, the application of the mTyr-derived hydrogels for the release of bioactive compounds should be demonstrated. Given the good substrate specificity of tyrosinase, such bioactive molecules may be incorporated prior to hydrogel synthesis in case they do not bear groups oxidized by tyrosinase or are sensitive to accompanying enzymes in the mTyr supply.^87^ Alternatively, slow loading by diffusion of bioactives into preformed gels may be applied. Here, heparin (*M*_n,GPC_ 36 kDa, polydispersity 1.3) was selected and incorporated in the reaction mixture of DAT-sOEG 10 kDa hydrogels prior to synthesis, in which the concentration of polymer was varied between 1.8, 3.2, and 7.2 wt %. Controllable release kinetics with a quantitative heparin release after 24 h, 32 h, and 5 d, respectively, were observed (Figure 10A). Fitting the initial phase of the release profiles illustrated that compound release is proportional to t^0.5^ (Figure 10B), supporting that the synthesized hydrogel networks are suitable for a diffusion-controlled mass transport with tailorable rates.

**Figure 10:**
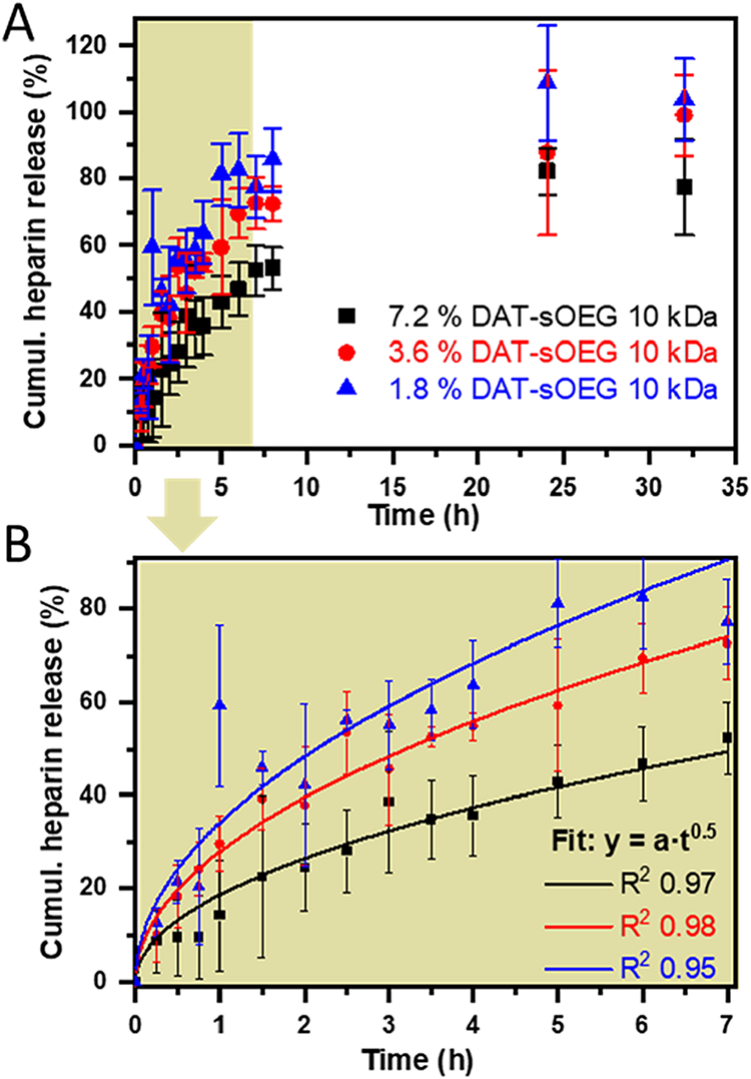
Release of heparin from flat film hydrogel samples depending on the DAT-sOEG 10 kDa precursor concentration used during synthesis with 1000 U·mL^–1^ mTyr. (A) Overall release profile. (B) Fitting of the initial phase of the release profile with a power law, using 0.5 as the exponent, as applicable for Case 1 transport (Fickian diffusion) from thin films. Error bars represent the standard deviation between the quantified heparin amounts released from independent samples (*n* = 4).

PEG-based biomaterials are known for their stealth properties in a biological context,^15^ i.e., they have inert interfaces that are not easily modified by protein attachment or cell adhesion and show biocompatibility. To evaluate whether the artificial substrates, the use of tyrosinase, or the formed networks/netpoints behave as expected, a set of biological experiments was conducted with DAT-sOEG 10 kDa hydrogels. L929 cells, commonly used for standardized cytotoxicity testing either in direct contact or via material extracts according to ISO 10993–5, were chosen as an example of an adherent cell line. THP-1 cells and THP-1 Blue cells, which are human monocyte cell lines, were additionally studied as representatives of nonadherent cells with biological functions of the innate immune system.^88^ Direct contact testing was preferred as this represents the more challenging condition compared to cell incubation with material extracts.^89^ L929 cells demonstrated a low cytotoxicity of the free telechelic DAT-sOEG (Figure 11A). A reduced viability in the presence of free mTyr is in line with observations from literature,^90^ while the exposure to the total mTyr as free enzyme would not occur in a realistic biological setting of hydrogel application. Furthermore, DAT-sOEG gels confirmed their biological inertness, which translated into nonadherence of L929 cells and thus an associated reduced proliferation and viability relative to untreated control cells. Known strategies to address this phenomenon of PEG-based hydrogels with adherent cells are the patterning of gel surfaces or the incorporation of cell-binding motifs into the hydrogels,^91–93^ which may be followed up in future studies. Importantly, excellent cell viability was demonstrated for THP-1 Blue cells as nonadherent cells, suggesting that neither the final hydrogels nor their components induced cytotoxicity in monocytes (Figure 11B).

**Figure 11:**
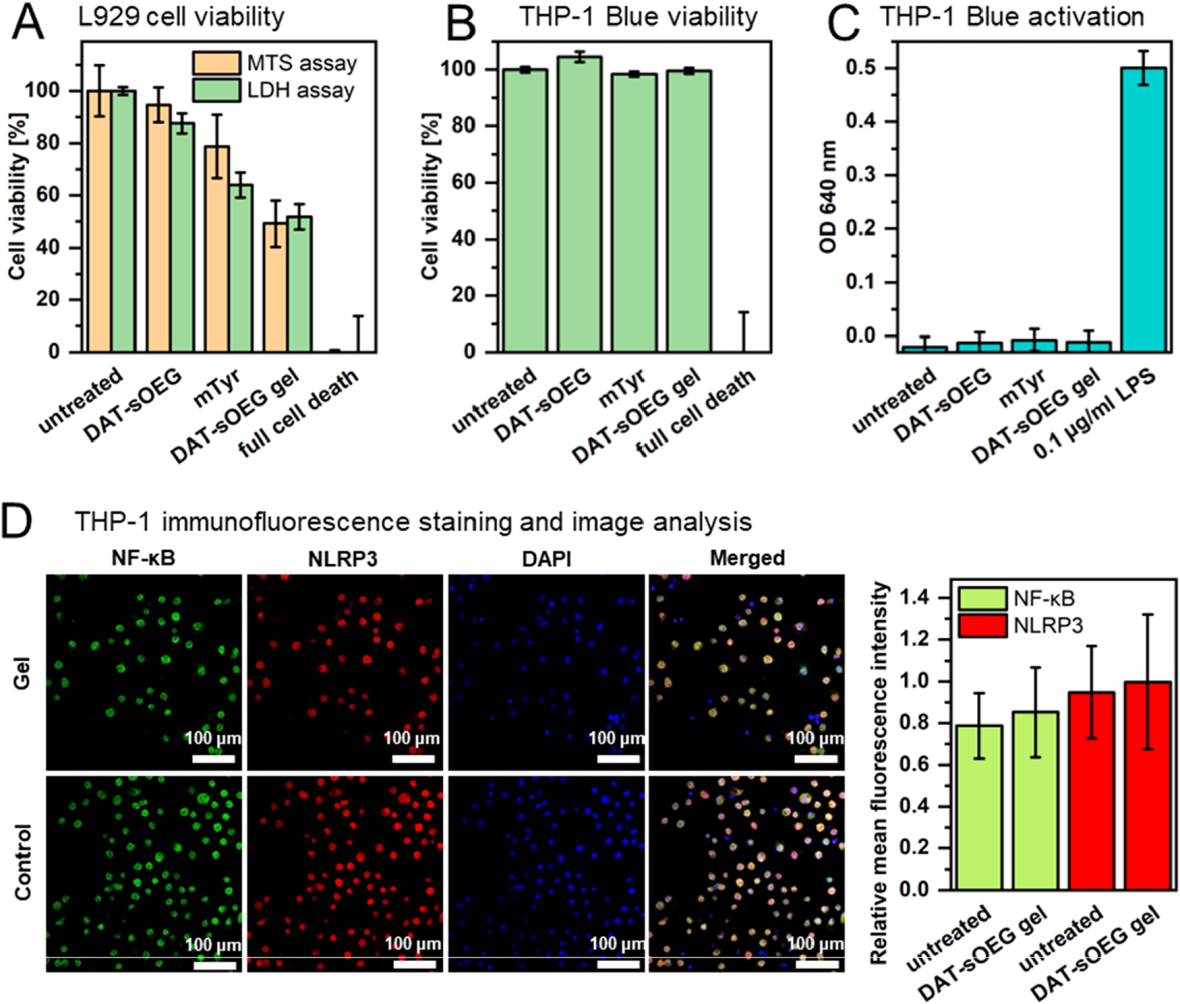
Biological evaluation of DAT-sOEG 10 kDa hydrogels (direct contact testing). (A) Cell viability of L929 cells after 48 h of incubation on the gels or with equivalent amounts of soluble mTyr or DAT-sOEG telechelics, assessed by LDH and MTS assays (*n* = 24, mean, S.D.). (B) Cell viability of THP-1 Blue cells after 24 h of incubation (LDH assay) (*n* = 18, mean, S.D.). (C) NF-κB–dependent SEAP expression in THP-1 Blue cells after 24 h of incubation. LPS served as a positive control (*n* = 18, mean, S.D.). (D) Investigation of colocalization of NF-κB and NLRP3 in THP-1 cells by confocal fluorescence microscopy. Images from a single z-plane showing the expression of NF-κB (green) and NLRP3 (red), as well as the DAPI staining of the nucleus (blue). Bar graphs represent the relative mean fluorescent intensity of NF-κB and NLRP3 (normalized to the DAPI MFI for each image) (*n* = 3, mean, S.D.).

THP-1 Blue cells are equipped with a reporter gene that enables the detection of immunoactivation in response to various stimuli via the nuclear factor-kappa B (NF-κB) pathway. Using the QuantiBlue assay, it was verified that NF-κB activity levels were comparable to those of the control group, while the known immunostimulator LPS induced a strong response (positive control) (Figure 11C). To further test whether pro-inflammatory signaling through NOD-like receptor family pyrin domain-containing 3 (NLRP3) inflammasome or NF-κB signaling in monocytes may be influenced by the hydrogels, immunofluorescence staining was performed. In the DAT-sOEG-10 kDa hydrogel group, NLRP3 and NF-κB levels were comparable to those observed in control cells (Figure 11D). Furthermore, flow cytometry was used to investigate whether DAT-sOEG-10 kDa hydrogels influence the immunophenotypic characteristics of the cells, as monocytes can shift phenotype or differentiate into macrophages under certain conditions. CD45 expression levels were identical to those of untreated cells, and only a slight increase in CD86 expression was observed (Figure 12). Similarly, CD11c levels were unchanged and CD206 expression was slightly increased (Supporting Figure 11). Overall, no pronounced changes in the expression of monocyte activation/differentiation markers or costimulatory molecules were observed, indicating that the DAT-sOEG hydrogels do not exert detrimental immunological effects under the applied direct-contact testing conditions.

**Figure 12:**
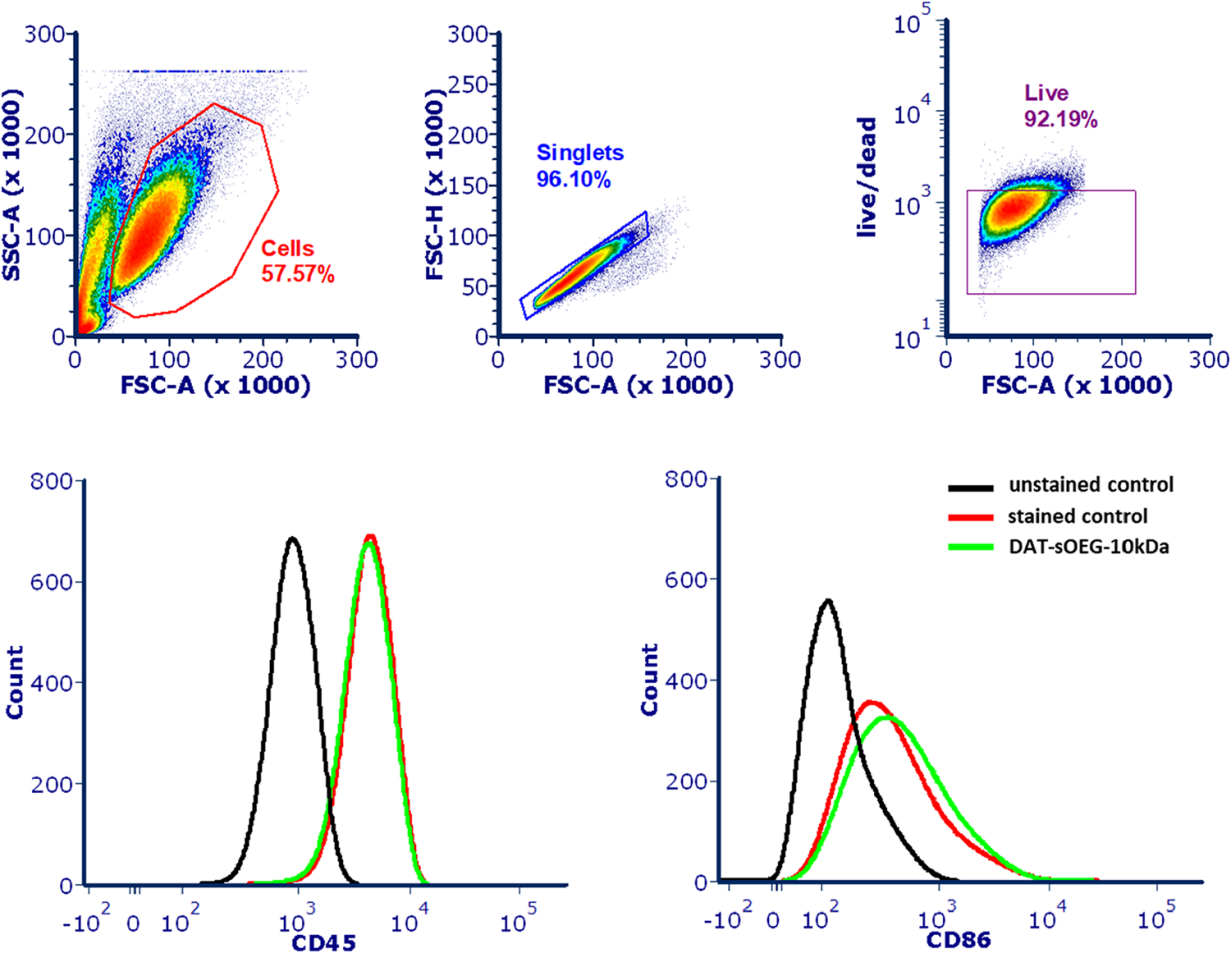
Immunophenotypic characterization of THP-1 cells after direct exposure to DAT-sOEG 10 kDa hydrogels. Top row: Gating strategy. Bottom row: Representative expression pattern for CD45 and CD86. More details are shown in Supporting Figure 11.

## 4 Conclusions

In this study, DAT and DATT were introduced as artificial substrates for mTyr and enabled a green approach to synthesize hydrogel network materials by enzymatic catalysis. These materials were also suitable for the incorporation and release of macromolecules at tunable rates depending on the hydrodynamic size of the payload or the network density. A set of first biological tests confirmed that the mTyr catalyzed synthesis of the hydrogels produced nonadhesive materials with no detrimental effects on monocyte activation or polarization toward macrophages. Some further remarkable findings of this study include the fact that the artificial monophenolic substrates DAT and DATT showed improved conversion rates by mTyr compared to the natural substrate tyrosine, which opens a field for a more detailed exploration of substrate binding and conversion in the future. Furthermore, a major focus of this work was to address the highly challenging task of better understanding the kinetics of reaction, the formed intermediates, and the structural motifs of products (netpoint motifs) resulting from the spontaneous and competitive cross-linking reactions that continue after mTyr-based substrate oxidation. Through model reactions and various analytical techniques, an understanding, e.g., of predominant reaction paths, the functionality of formed netpoints, and the structural composition of complex mixtures of cross-links could be gained. Overall, beyond the results reported here, this study may serve as a template to evaluate not only the properties of enzyme-catalyzed hydrogels, but also for gaining insights into their molecular architecture and chemistry of cross-links as necessary to foster the (clinical) translation of hydrogel networks cross-linking by enzyme-catalyzed, green chemistry approaches.

## Supplementary Material



## Notes

MD simulation of ligand interaction with mTyr in the presence of water; docking poses, RMSD profiles, and distance to His85 for OEG_6_-conjugated substrates; MD simulation for solvent accessible surface areas for DAT-sOEG_6_ and DATT-sOEG_6_; FTIR spectroscopy demonstrating the coupling of DAT to sOEG; wavelength of maximum absorption λmax of model phenols, catechols, quinones, and coupled structures; UHPLC-ESI-MS analysis to assess the degree of DAT-lmOEGOMe 2 kDa conversion by mTyr in model reactions; ^1^H NMR analysis of model reactions of netpoint formation; Immunophenotypic characterization of THP-1 cells after direct contact with DAT-sOEG 10 kDa hydrogels. Tables: Interpretation of netpoint structures in model reaction of mTyr with DAT methyl ester (MALDI-ToF MS analysis); Prediction of ^1^H NMR signals for potential intermediate and netpoint structures; Mesh sizes ξ of hydrogels from DAT­(T)-sOEG depending on mTyr concentration, nature of substrate (DAT/DATT), and sOEG molecular weight (PDF)

## ∥

Institute of Materials Chemistry, Brandenburgische Technische Universität Cottbus-Senftenberg, 01968 Senftenberg, Germany
