# Supplementary material for: Tyrosinase Cross-Linked PEG Hydrogels with DAT and DATT as Artificial Substrates: Design, Structure, and Functions
Source: Biomacromolecules. 2026 Jan 26;27(2):1317–36. doi: 10.1021/acs.biomac.5c01929 (PMC12892322; doi:10.1021/acs.biomac.5c01929)
Supplement: Supplementary file 1 [file bm5c01929_si_001.pdf]

SUPPORTING INFORMATION

Tyrosinase crosslinked PEG hydrogels with DAT and DATT as artificial substrates: Design, structure, and functions

Miroslava Racheva, Javier Basalo Lourido, Enise Ece Gurdal, Martin Herbst, Seyhmus Bayar, Daniela Radzik, Elen Bähr, Constanze Zwies, Axel T. Neffe, Markus Pietzsch, Andreas Lendlein, Christian Wischke*

* Correspondence: C. Wischke, E-mail: christian.wischke@pharmazie.uni-halle.de

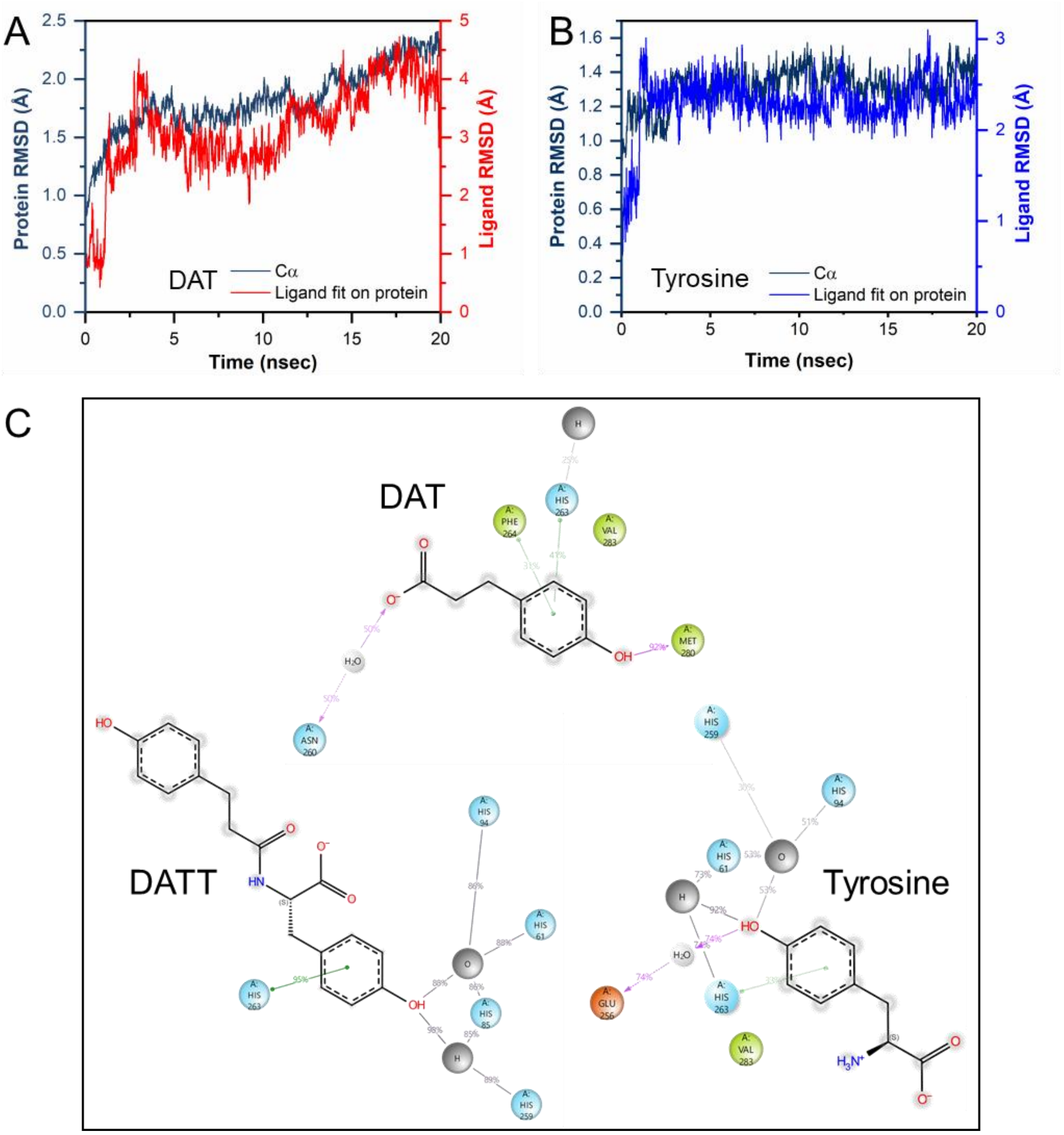

Supp. Fig. 1: MD simulation of ligand interaction with mTyr in the presence of water. (A-B) RMSD analysis of protein backbone and DAT (A) as well as tyrosine (B) over 20 ns MD simulations, which indicates stable binding of DAT and tyrosine to mTyr.

(C) Ligand interaction diagrams from MD simulations showing key binding interactions between mTyr and each ligand. The gray spheres labelled as O and H correspond to the hydroxide ion in the active center. All three ligands, DAT, DATT, and tyrosine, engage in π–π stacking with His263 via their phenolic ring. The main interactions are shown, which occurred more than 25% (DAT), 80% (DATT), 30% (tyrosine) of the simulation time in the selected trajectory (0.00 through 20.02 nsec).

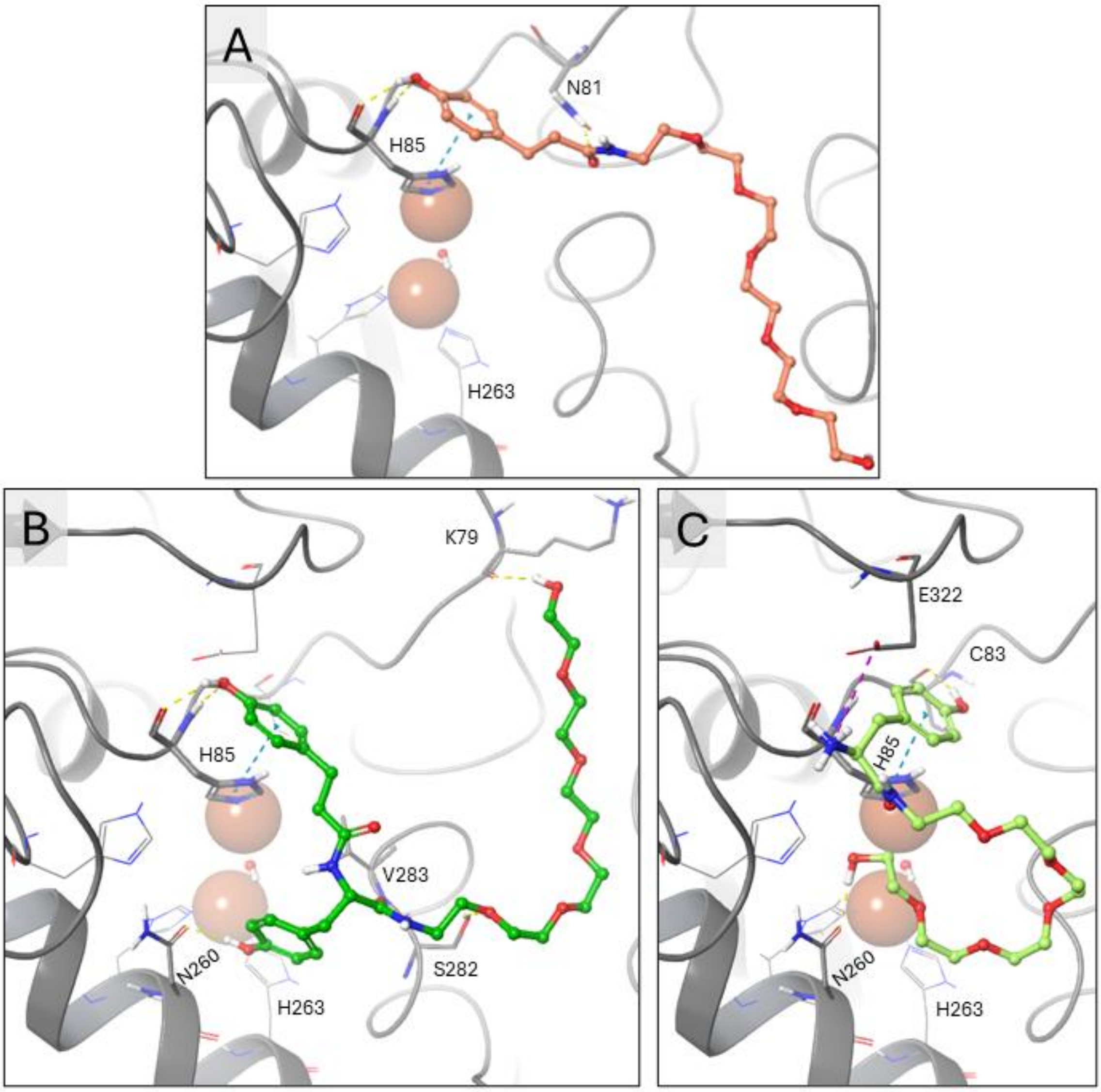

Supp. Fig. 2: Docking poses for (A) DAT-OEG$_6$ (docking score: -4.66), (B) DATT-OEG$_6$ (docking score: -7.08), (C) tyrosine-OEG$_6$ (docking score: -3.34) at the mTyr binding site (PDB ID: 2Y9W). Hydrogen bonds are depicted as yellow dashed lines, and π-π stacking interactions are shown as cyan dashed lines.

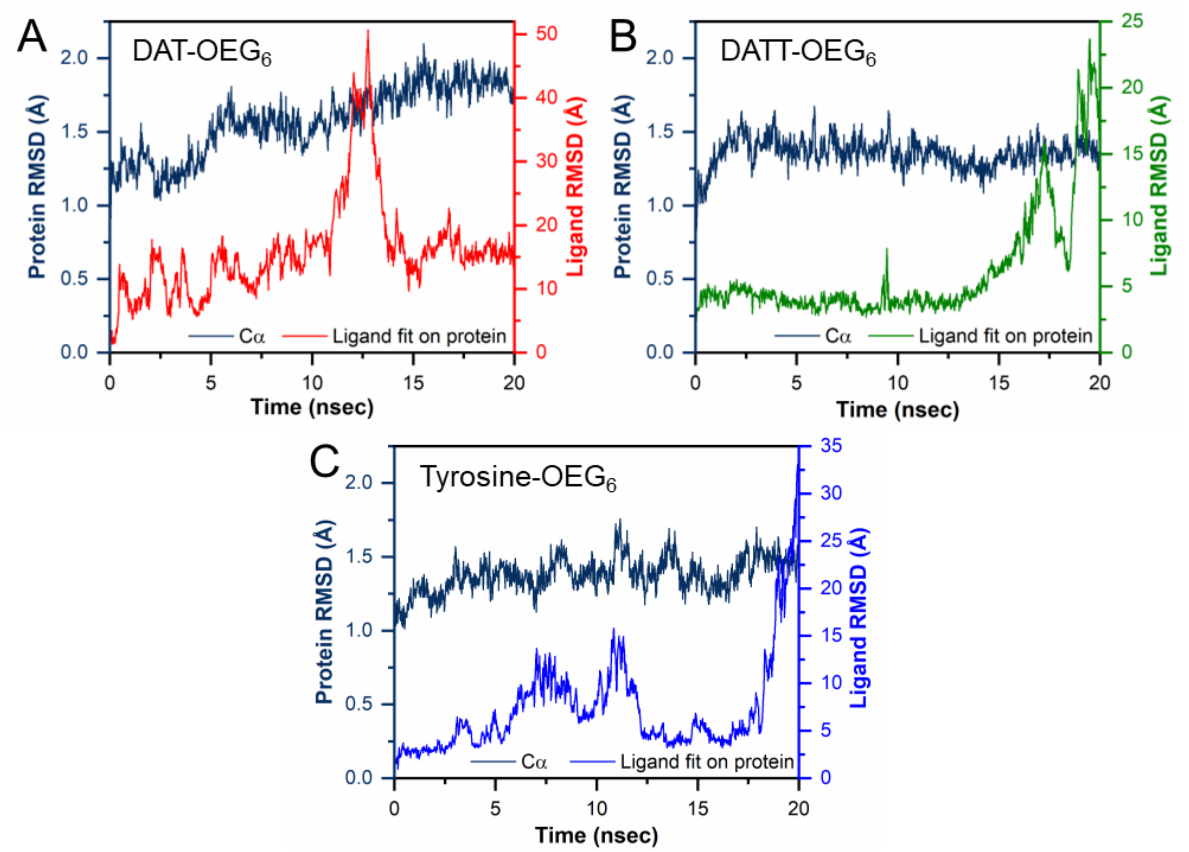

Supp. Fig. 3: RMSD profiles of OEG_6-conjugated (A) DAT, (B) DATT, and (C) tyrosine bound to mTyr over 20 ns MD simulations. The protein backbone RMSD remained well-converged throughout the simulations, confirming that the bulky OEG_6 scaffolds did not perturb the overall structure of mTyr. The RMSD values (fit on the protein) of pegylated substrates were substantially higher for the OEG_6 systems compared to the free substrates (Supp. Fig. 1), which can be attributed to the pronounced conformational flexibility of the OEG_6 chains rather than to ligand dissociation.

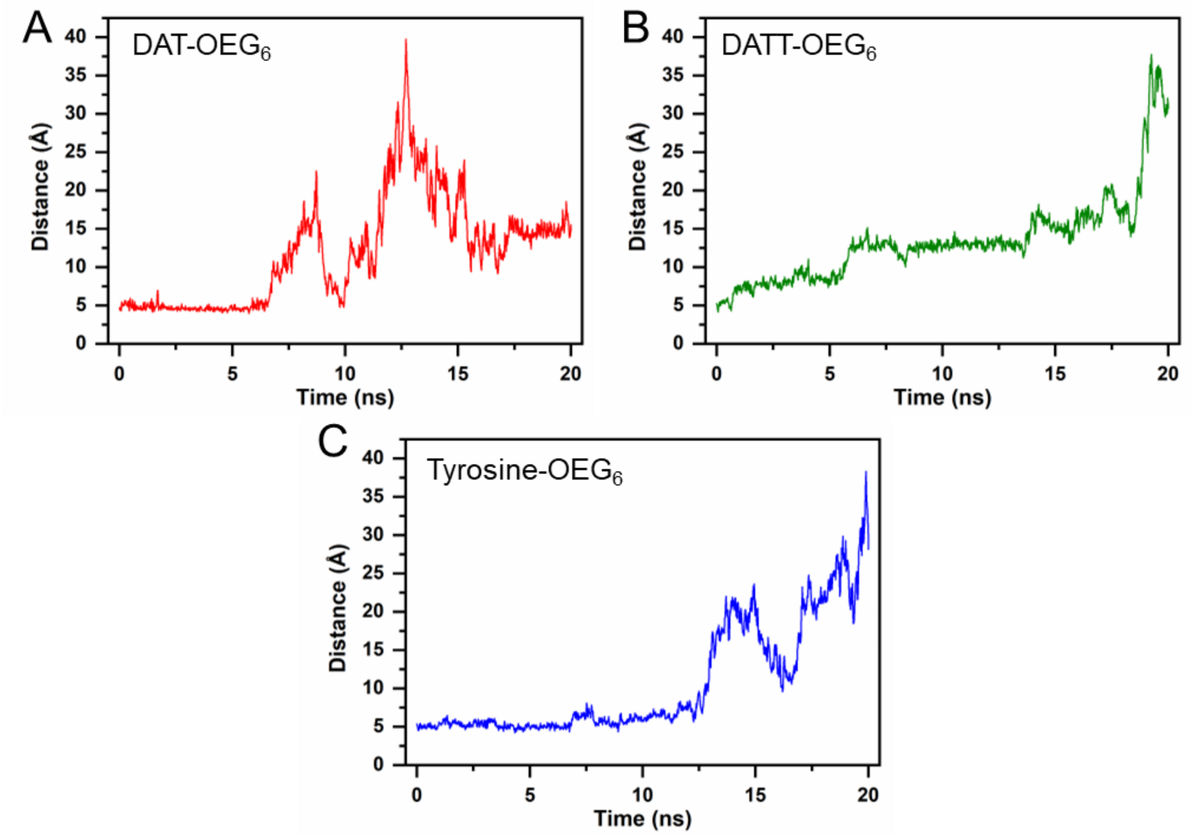

Supp. Fig. 4: Distances between the His85 of the mTyr active center and the phenolic ring of (A) OEG_6 conjugated DAT, (B) OEG_6 conjugated DATT, and (C) OEG_6 conjugated tyrosine over 20 ns MD simulations. The observations suggest that increasing molecular bulk and flexibility through dual phenolic substitution and PEGylation progressively decreases the residence time of the reactive ring system within the His85/CuA coordination region, while a productive positioning (low distance to His85) of the substrate is given in the active site over parts of the simulation.

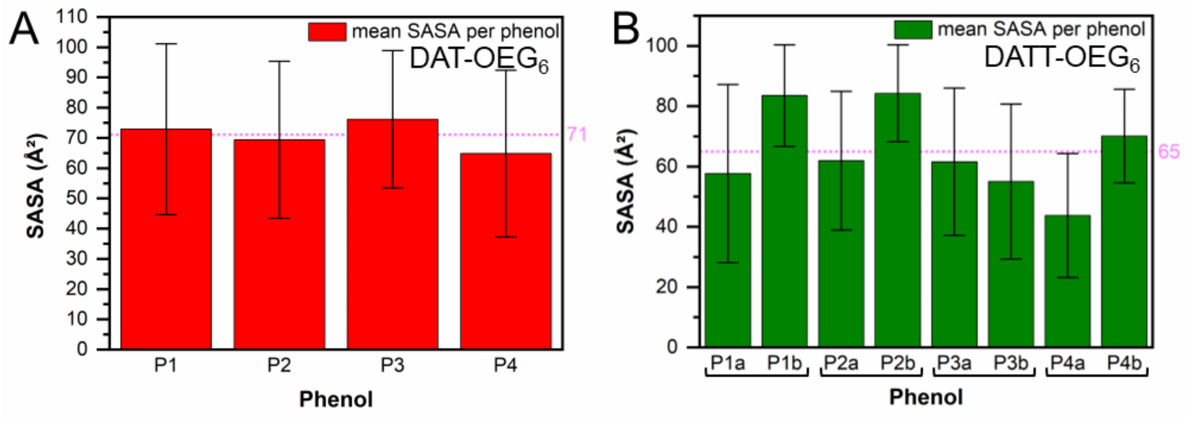

Supp. Fig. 5: MD simulation of solvent accessible surface areas (SASA) of the phenolic moieties in sOEG6 conjugated substrates for (A) DAT-sOEG6 and (B) DATT-sOEG6. The simulation was performed over 20 ns, showing mean and S.D. Dashed lines indicate overall mean. Phenol groups of DATT-sOEG were distinguished according to docking pose in Supp. Fig. 2 as Pa (His85-interacting) and Pb for clarity.

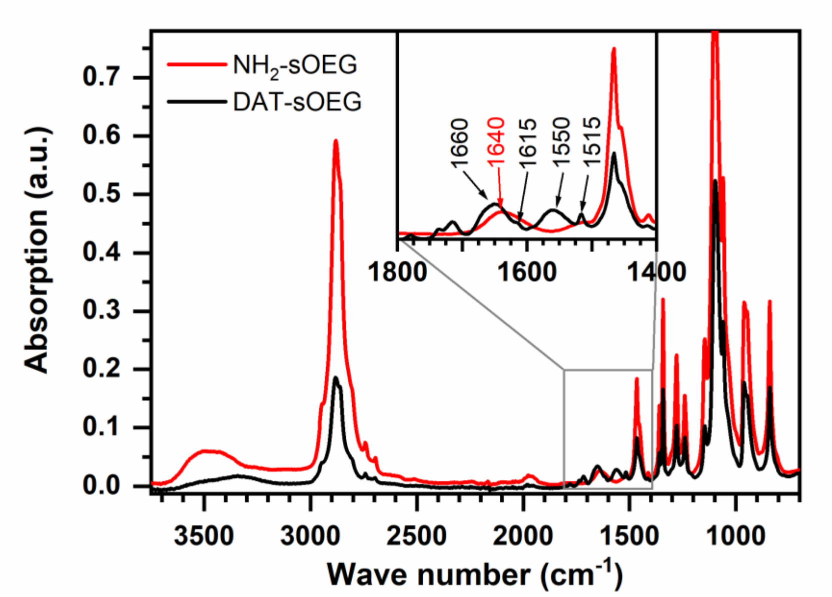

Supp. Fig. 6: FTIR spectroscopy demonstrating the coupling of DAT to sOEG via EDC/NHS chemistry. Data for the non-functionalized starting material, sOEG with amino end groups, and DAT-functionalized sOEG.

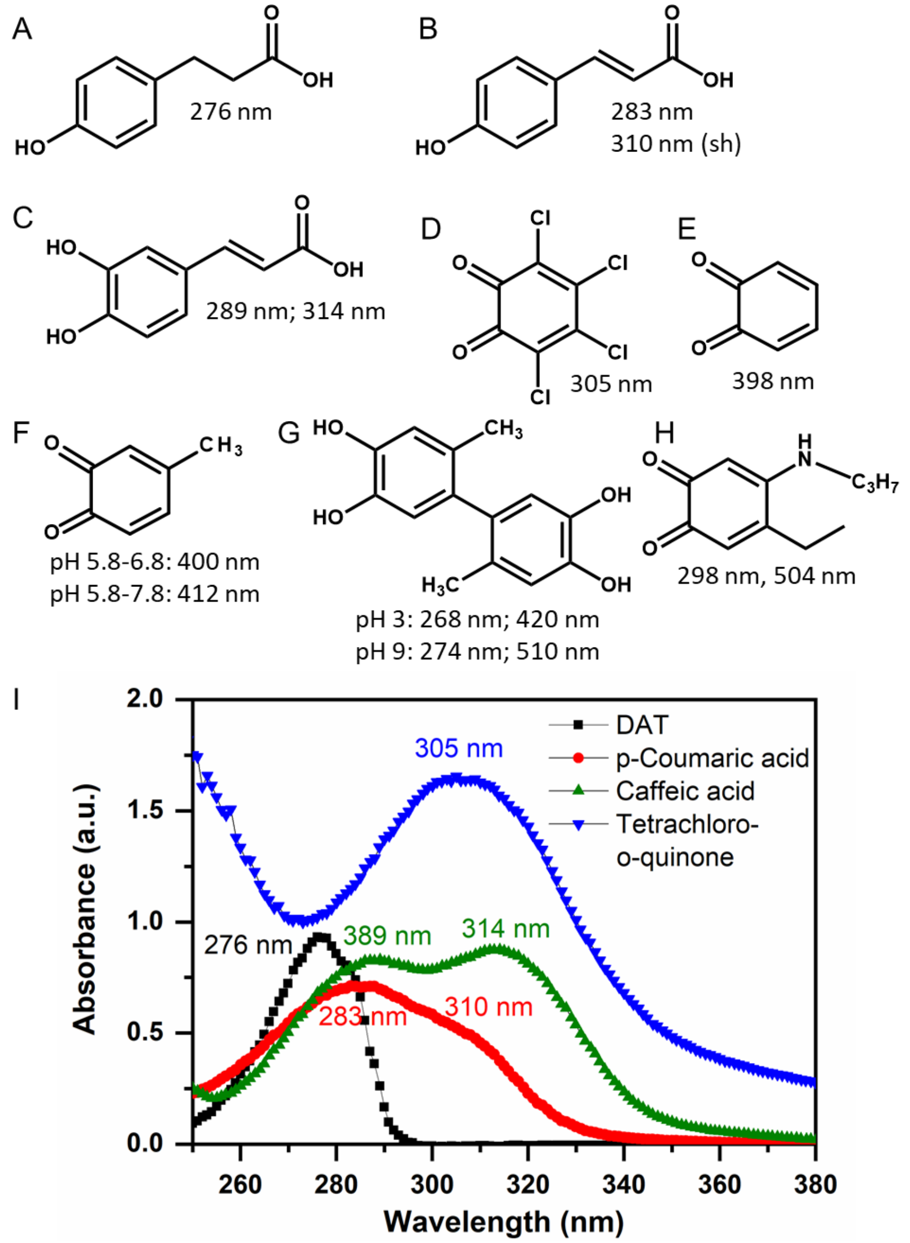

Supp. Fig. 7: Wavelength of maximum absorption λ_{max} of model phenols, catechols, quinones, and coupled structures. Measured reference substances: (A) DAT, (B) p-coumaric acid, (C) caffeic acid, (D) tetrachloro-*o*-quinone. Further data from literature: (E) 1,2-quinone [J. Phys. Chem. A 114 (28): 7470-7478 2010], (F) 4-methyl-1,2-quinone [Anal. Biochem. 75, 211-218 1976], (G) di-DOPA adducts [Biochim Biophys Acta 1118(2): 134-138 1992], (H) quinone-amine adduct [Science 273(5278): 1078-1084, 1996]. (I) UV absorbance spectra for (A) at 100 µg/ml, (B) at 5 µg/ml, (C) at 12 µg/ml, and (D) as saturated solution. The abbreviation (sh) stands for shoulders occurring along with the main peak.

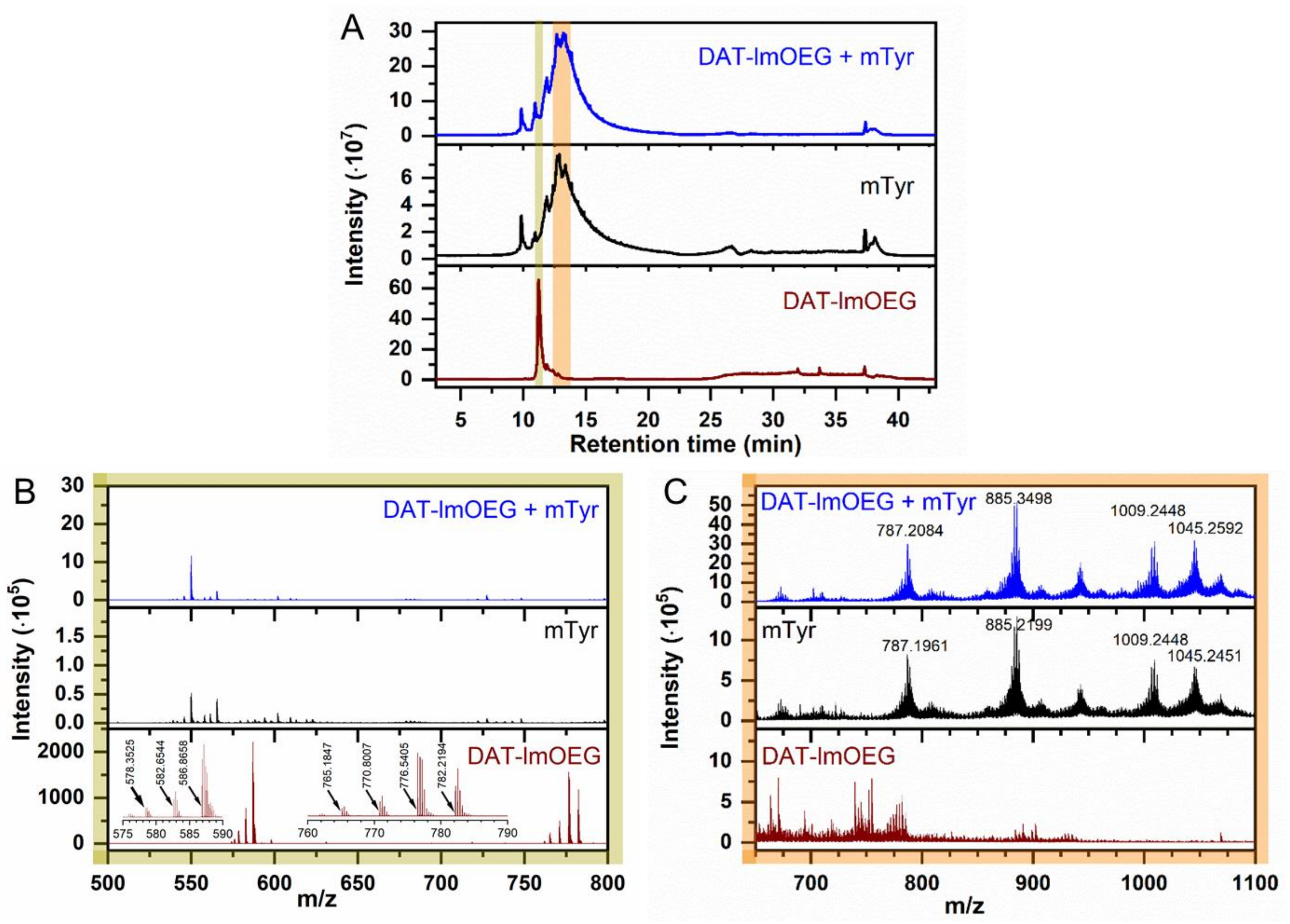

Supp. Fig. 8: UHPLC-ESI-MS analysis to assess the degree of DAT-lmOEG$_{OMe}$ 2 kDa conversion by mTyr in model reactions. (A) Total ion chromatograms (TIC) trace of the chromatographic separation on a C-18 column for the reaction mixture of

DAT-lmOEG$_{OMe}$ (5 mg·ml^{-1}, d.f. 82%) + mTyr (1700 U·ml^{-1}) after 24 h, pure mTyr, and pure DAT-lmOEG$_{OMe}$ 2 kDa. (B) Average mass spectra at r.t. 11.1-11.3 min corresponding to the position of the main chromatographic signal of DAT-lmOEG in panel (A). Insets for DAT-lmOEG$_{OMe}$ 2 kDa show labeled monoisotopic molecular ions assigned as $[M+3H+NH_4]^{4+}$ (m/z 578.4053, Δm/z 0.0528), $[M+2H+2(NH_4)]^{4+}$ (m/z 582.6544, Δm/z 0.0453), $[M+H+3(NH_4)]^{4+}$ (m/z 586.9189, Δm/z 0.05311) as well as $[M+3H]^{3+}$ (m/z 765.1847, Δm/z 0.0595), $[M+2H+NH_4]^{3+}$ (m/z 770.8582, Δm/z 0.0575), $[M+H+2(NH_4)]^{3+}$ (m/z 776.5405, Δm/z 0.0642) and $[M+3(NH_4)]^{3+}$ (m/z 782.2194, Δm/z 0.0676). (C) Average mass spectra at r.t. 12.4-13.7 min selected from the broad chromatographic signal of mTyr and DAT-lmOEG+mTyr in panel (A). The MS spectra of panel (B) show the conversion of DAT-lmOEG$_{OMe}$ educts based on the disappearance of the respective signals after reaction with mTyr. The MS spectra of panel (C) were dominated by broad signals of mTyr (indicated m/z represents the most intense signal of a respective group of signals) and are not indicative of products of the model reaction.

Supp. Tab. 1: Interpretation of netpoint structures in model reaction of mTyr with DAT methyl ester (MALDI-ToF MS analysis).

	**Experimental data**				**Mass matches**	
	Peak ID	m/z (pos. mode)	S/N ratio	Rel. AUC (%)	Structural elements	Δ m/z
Trimer region	1	591.397	14	26	(Q+C+CA-4H)+Na$^+$ (*)	+0.249
					(2C+P-4H)+Na$^+$	+0.219
	2	593.397	13	25	(2C+CA-4H)+Na$^+$	+0.239
	**3**	**605.382**	**40**	**84**	**(Q+2C-4H)+Na$^+$**	**+0.224**
	**4**	**607.4**	**74**	**100**	**(3C-4H)+Na$^+$**	**+0.232**
					(2C+P-4H)+K$^+$	+0.247
	5	621.399	9	40	(Q+C+HC-4H)+Na$^+$ (**)	+0.241
					(Q+2C-4H)+K$^+$	+0.267
	6	623.411	10	19	(2C+HC-4H)+Na$^+$	+0.243
					(3C-4H)+K$^+$	+0.269
	7	639.438	29	64	(2C+HC-4H)+K$^+$	+0.296
	8	661.404	8	18	*not assigned*	–
Tetramer region	9	783.424	19	55	(2Q+C+CA-6H)+Na$^+$ (*)	+0.214
					(Q+2C+P-6H)+Na$^+$	+0.201
					(Q+3C-6H-O)+Na$^+$ (***)	+0.206
	10	785.416	20	58	(Q+2C+CA-6H)+Na$^+$ (*)	+0.214
					(4C-6H-O)+Na$^+$ (**)	+0.188
	11	787.458	19	60	(3C+CA-6H)+Na$^+$	+0.214
	12	789.464	10	33	*not assigned* (****)	–
	13	797.404	9	24	(2Q+2C-6H)+Na$^+$	+0.201
	**14**	**799.42**	**25**	**70**	**(Q+3C-6H)+Na$^+$**	**+0.207**
					(Q+2C+P-6H)+K$^+$	+0.214
	**15**	**801.437**	**26**	**77**	**(4C-6H)+Na$^+$**	**+0.214**
					(3C+P-6H)+K$^+$	+0.214
	16	815.443	24	70	(Q+2C+HC-6H)+Na$^+$ (**)	+0.230
					(Q+3C-6H)+K$^+$	+0.256
	17	817.442	15	45	(3C+HC-6H)+Na$^+$	+0.245
					(4C-6H)+K$^+$	+0.245
	18	831.448	7	20	(Q+2C+HC-6H)+K$^+$(**)	+0.261
Pentamer region	19	963.46	6	16	(2Q+1C+2CA-8H)+Na$^+$ (*)	+0.223
					(Q+3C+CA-8H-O)+Na$^+$ (* / ***)	+0.198
					(Q+2C+CA+P-8H)+Na$^+$ (*)	+0.193
	20	965.47	7	21	(Q+2C+2CA-8H)+Na$^+$ (*)	+0.223
					(4C+CA-8H-O)+Na+ (* / ***)	+0.198
					(Q+2C+CA+P-8H)+Na$^+$ (*)	+0.193
	21	977.476	11	32	(2Q+2C+CA-8H)+Na$^+$ (*)	+0.229
					(Q+4C-8H-O)+Na$^+$ (***)	+0.204
					(Q+3C+P-8H)+Na$^+$	+0.199
	22	979.483	14	42	(Q+3C+CA-8H)+Na$^+$ (*)	+0.226
					(5C-8H-O)+Na$^+$	+0.201
	23	981.489	10	31	(4C+CA-8H)+Na$^+$	+0.222
	24	991.473	12	33	(2Q+3C-8H)+Na$^+$	+0.216
					(2Q+P+2C-8H)+K$^+$	+0.232
	**25**	**993.487**	**23**	**68**	**(Q+4C-8H)+Na$^+$**	**+0.220**
					(Q+P+3C-8H)+K$^+$	+0.236
	**26**	**995.49**	**19**	**56**	**(5C-8H)+Na$^+$**	**+0.213**
					(P+4C-8H)+K$^+$	+0.229
	27	1009.518	12	35	(Q+3C+HC-8H)+Na$^+$ (**)	+0.251
					(Q+4C-8H)+K$^+$	+0.277
	28	1011.52	10	31	(4C+HC-8H)+Na$^+$	+0.243
					(5C-8H)+K$^+$	+0.269
Hexamer region	29	1157.558	7	18	(2Q+3C+CA-10H)+Na$^+$ (*)	+0.266
					(Q+4C+CA-10H-O)+Na$^+$ (* / ***)	+0.241
					(Q+3C+CA+P-10H)+Na$^+$ (*)	+0.236
	30	1159.565	8	19	(Q+3C+2CA-10H)+Na$^+$ (*)	+0.263
					(5C+CA-10H-O)+Na$^+$ (* / ***)	+0.238
					(4C+CA+P-10H)+Na$^+$	+0.233
	31	1171.577	12	31	(2Q+3C+CA-10H)+Na$^+$(*)	+0.275
					(Q+5C-10H-O)+Na$^+$ (***)	+0.250
					(Q+4C+P-10H)+Na$^+$	+0.245
	32	1173.581	14	37	(Q+4C+CA-10H)+Na$^+$(*)	+0.269
					(6C-10H-O)+Na$^+$(***)	+0.244
					(5C+P-10H)+Na$^+$	+0.239
	33	1175.583	10	27	(5C+CA-10H)+Na$^+$	+0.261
	34	1185.578	11	27	(2Q+4C-10H)+Na$^+$	+0.266
					(2Q+3C+P-10H)+K$^+$	+0.282
	**35**	**1187.59**	**17**	**45**	**(Q+5C-10H)+Na$^+$**	**+0.268**
					(Q+4C+P-10H)+K$^+$	+0.284
	36	1189.593	14	38	(6C-10H)+Na$^+$	+0.261
					(5C+P-10H)+K$^+$	+0.277
	37	1201.606	7	16	(2Q+3C+HC-10H)+Na$^+$ (**)	+0.294
					(2Q+4C-10H)+K$^+$	+0.320
	38	1203.618	12	28	(Q+4C+HC-10H)+Na$^+$ (**)	+0.296
					(Q+5C-10H)+K$^+$	+0.322
Heptamer region	39	1365.668	7	14	(2Q+4C+CA-12H)+Na$^+$	+0.312
					(Q+6C-12H-O)+Na$^+$	+0.287
					(Q+5C+P-10H)+Na$^+$	+0.282
	40	1379.683	7	14	(2Q+5C-12H)+Na$^+$	+0.317
					(2Q+4C+P-12H)+K$^+$	+0.333
	**41**	**1381.693**	**10**	**19**	**(Q+6C-12H)+Na$^+$**	**+0.317**
					(Q+5C+P-12H)+K$^+$	+0.333

Out of all detected signals, the apparently monoisotopic peaks with S/N ratio ≥ 6 are displayed and analyzed. Main peaks of a given spectrum region are printed in bold. Individual peaks are separated by dotted lines. Clusters of peaks are separated by dashed lines. Δ m/z represents the mass determined by MALDI-MS minus the monoisotopic mass of the assigned structure.

In principle, coupling of aryloxyl radicals can lead to carbon-carbon bridges or carbon-oxygen-carbon bridges. Product masses are lower than the sum of masses of the monomeric structural elements (e.g. Q, C, …) by a value corresponding to 2 protons (H) for each bond between multimer units e.g. at 2', 5' or 6' position of the six-membered rings (e.g. - 4H for trimers, -6H for tetramers).

Several experimentally observed masses may be assigned to a number of different adducts having similar m/z shifts, e.g. m/z shift + 39 might by water+ sodium adducts or potassium adducts. Grey lines correspond to less probable assignments as they e.g. represent asymmetric shifts to the group of main peaks or are potassium adducts that appear less prevalent given the dominant presence of sodium adducts in the starting materials.

* Demethylation of the methyl ester unit leads to free carboxyl groups. While here exemplarily indicated as CA structural element, the demethylation may also take place at a Q structural element instead, in case Q is part of the assigned structure.

** Hydration of Q leads to HC, which in principle can also react further to HQ (HC+ Q → HQ + C). While here exemplarily indicated as HC structural element, the hydrated structure may also be a HQ instead, in case Q is part of the assigned structure.

*** If a coupling between structural units proceeds at 3' or 4' position of the six-membered ring originally substituted with oxygen (keto group, hydroxyl group), a loss of oxygen at this position during the coupling reaction results in additional mass shift of m/z ~18.

**** Observed mass may be explained by (2C+2CA-6H)+K$^+$. This theoretical product would require a loss of two methyl groups and the presence of a K$^+$ adduct, which appears not to be highly probable based on other observed signals.

Supp. Table 2: Prediction of ^{1}H-NMR signals for potential intermediate and netpoint structures. Prediction in MestReNova software 12.0.2. does not allow to define specific solvents. Signal multiplicity considers higher order coupling (s singulett, d dublett, dd dublett of dublett, t triplett, m multiplett).

	Analyzed structure	Position	Signal multiplicity	Predicted chemical shift (ppm)
1	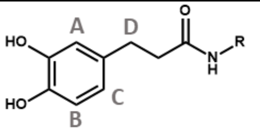	A	m	6.70
		B	m	6.72
		C	m	6.71
		D	t	2.83
2	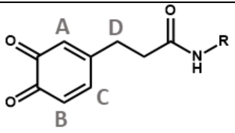	A	d	6.47
		B	d	6.40
		C	dd	7.06
		D	t	2.91
3	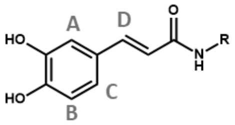	A	d	7.06
		B	d	6.79
		C	dd	7.02
		D	d	7.44
4	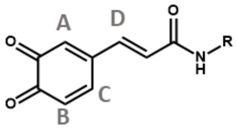	A	d	6.88
		B	d	6.81
		C	dd	7.62
		D	d	7.35
5	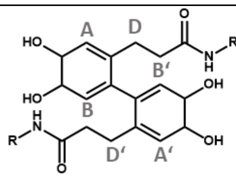	A/A‘	m	6.13
		B/B‘	m	6.52
		D/D‘	m	2.64; 2.69
6	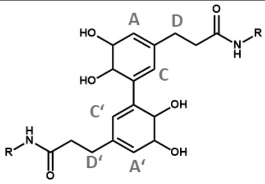	A/A‘	m	6.05
		C/C‘	m	6.59
		D/D‘	m	2.67; 2.70
7	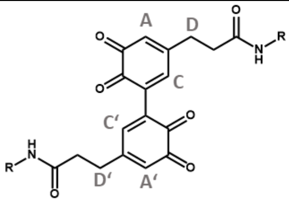	A/A‘	d	6.44
		C/C‘	d	7.17
		D/D‘	t	2.88
8	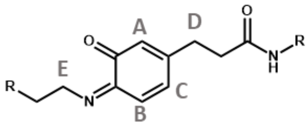	A	d	6.53
		B	d	7.31
		C	dd	7.11
		D	t	2.90
		E	t	4.21
9	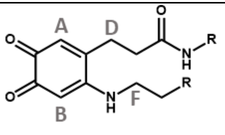	A	s	6.45
		B	s	6.43
		D	m	2.62
		F	m	3.25

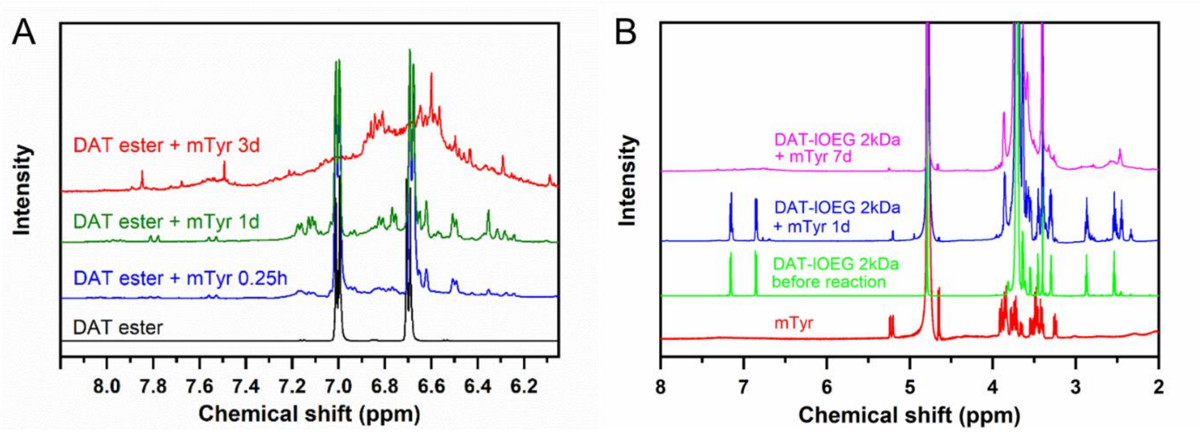

Supp. Fig. 9: ^{1}H-NMR analysis of model reactions of netpoint formation. (A) DAT methyl ester (2.5 mg·ml^{-1}) before and after 0.25 h, 1 d, and 3 d of incubation with mTyr (775 U·ml^{-1}) in D_2O saturated with O_2; spectra recorded in MeOH-d_4 after freeze drying. (B) DAT-lOEG$_{OMe}$ 2 kDa (3.6-5 wt.%) before and after 1 d and 7 d of incubation with mTyr (2200 U·ml^{-1}) in D_2O saturated with O_2; spectra recorded in D_2O.

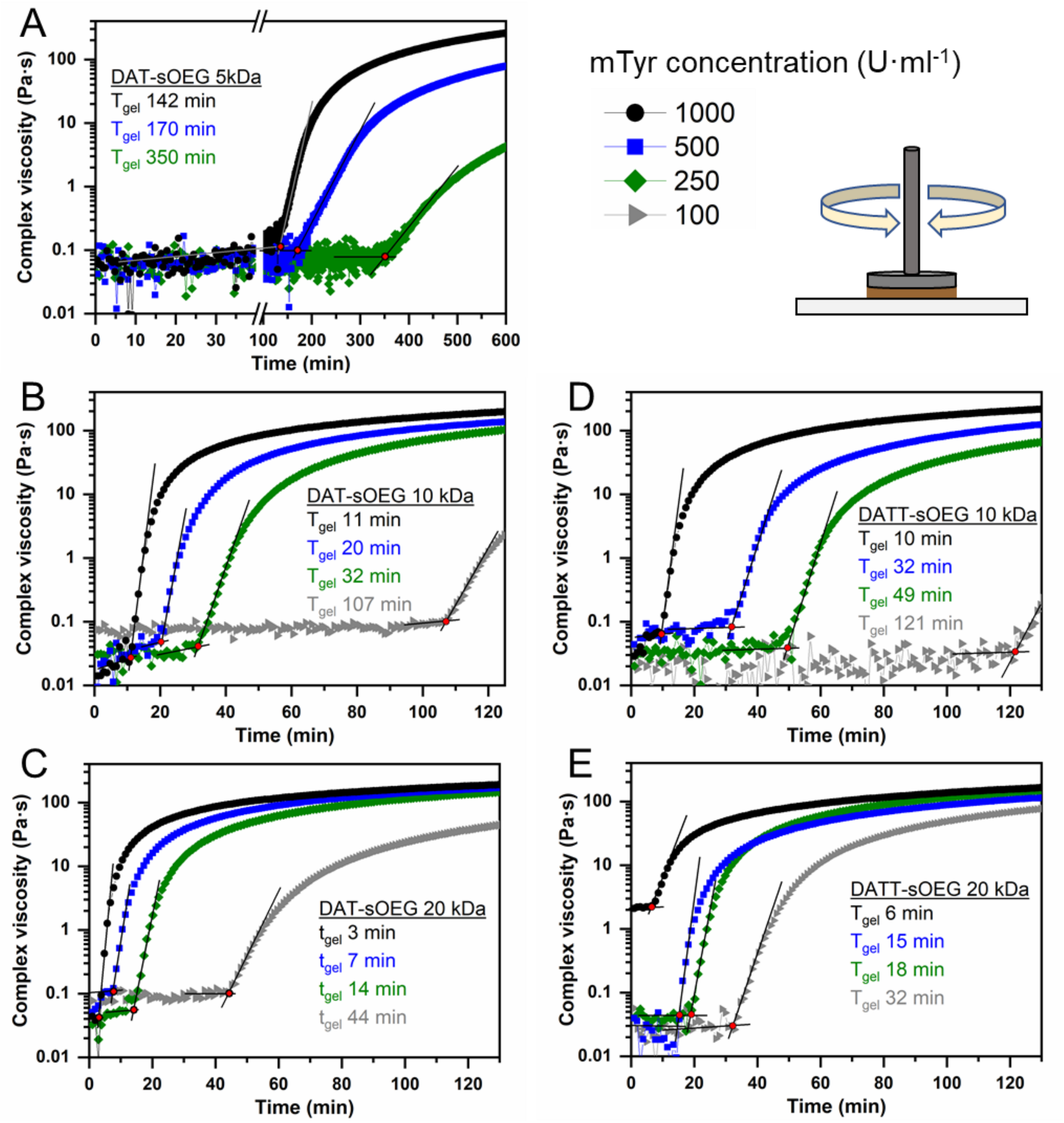

Supp. Fig. 10: Gelation kinetics of DAT- or DATT-sOEG by mTyr as investigated by oscillatory rheology. Data for (A) DAT-sOEG 5 kDa ($d.f._{NMR}$ 56 mol%), (B) DAT-sOEG 10 kDa ($d.f._{NMR}$ 61 mol%), (C) DAT-sOEG 20 kDa ($d.f._{NMR}$ 49 mol%), (D) DATT-sOEG 10 kDa ($d.f._{MALDI}$ 91 mol%), (E) DATT-sOEG 20 kDa ($d.f._{NMR}$ 55 mol%), each used at 5 wt.% with exposure to 100, 250, 500, or 1000 U·ml^{-1} mTyr in oxygen-saturated PBS buffer pH 7.4. The analysis was conducted at 25 °C.

Supp. Tab. 3: Mesh sizes ξ of hydrogels from DAT(T)-sOEG depending on mTyr concentration, nature of substrate (DAT/DATT), and sOEG molecular weight. ξ ± standard deviation was calculated according to rubber elastic theory from the elastic moduli of hydrogels, determined by oscillatory rheology at 37 °C (n ≥ 3).

	**ξ (nm)**			
Polymer type	**DAT-sOEG**		**DATT-sOEG**	
M_n (kDa)	mTyr conc. ($U{\cdot}mL^{-1}$)		mTyr conc. ($U{\cdot}mL^{-1}$)	
	100	500	100	500
5	15.6 ± 1.2 nm	16.5 ± 1.8 nm	n.d.*	n.d.*
10	13.3 ± 1.3 nm	12.2 ± 1.6 nm	13.6 ± 1.4 nm	15.5 ± 1.4 nm
20	13.4 ± 1.4 nm	12.5 ± 1.7 nm	13.3 ± 1.3 nm	12.6 ± 1.1 nm

** n.d. – not determined*

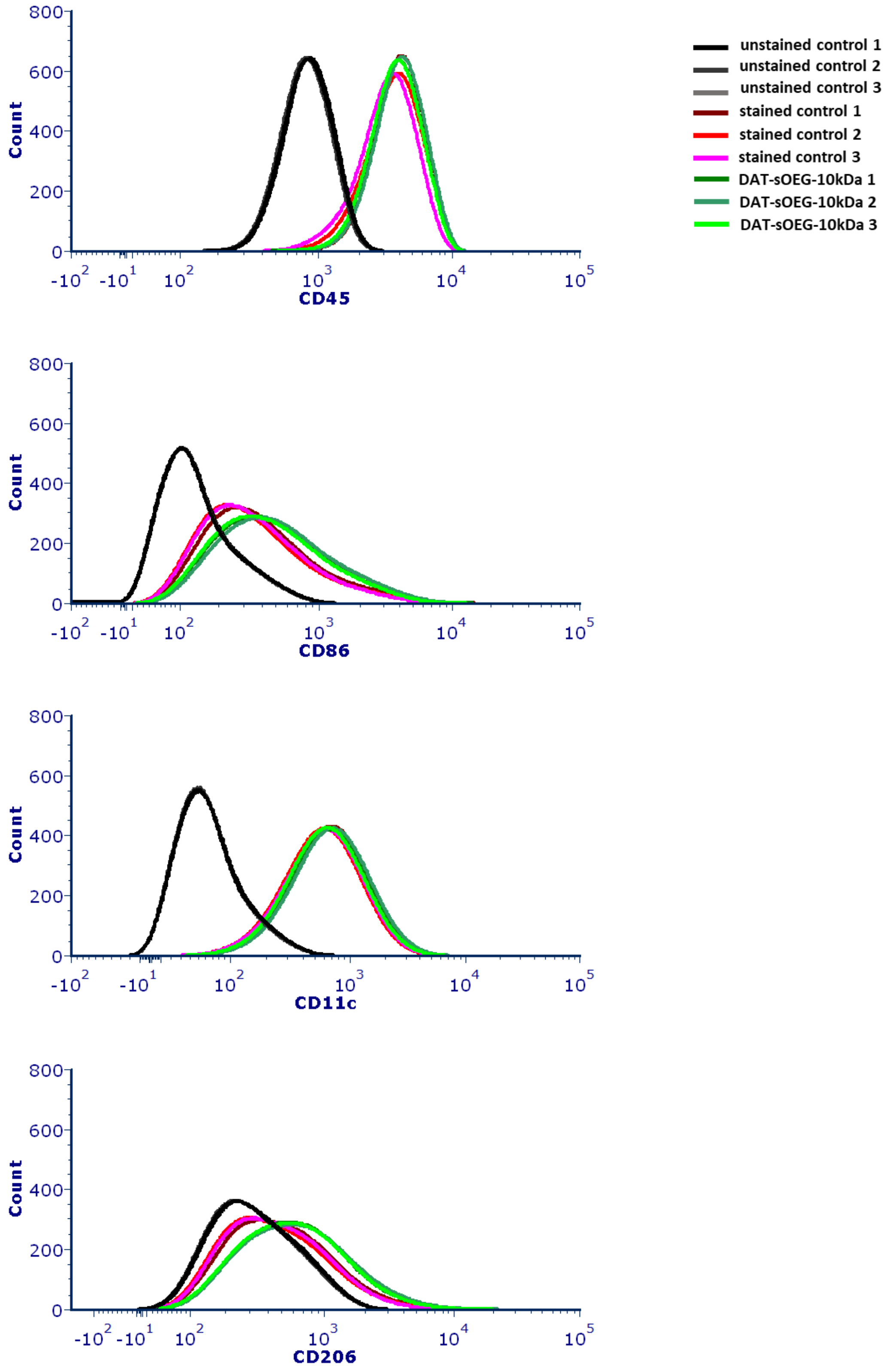

Supp. Fig. 11: Immunophenotypic characterization of THP-1 cells by flow cytometry after direct exposure to DAT-sOEG 10 kDa hydrogels, showing n=3 biological replicates for CD45, CD86, CD11c, and CD206 surface markers in viable cells.
